# Photodissociation Dynamics of the Highly Stable *ortho*-Nitroaniline Cation

**DOI:** 10.1021/acs.jpca.3c08364

**Published:** 2024-02-27

**Authors:** Hugo A. López Peña, Jacob M. Shusterman, Clayton Dalkiewicz, Shane L. McPherson, Christine Dunstan, Kunjal Sangroula, Ka Un Lao, Katharine Moore Tibbetts

**Affiliations:** Department of Chemistry, Virginia Commonwealth University, Richmond, Virginia 23284, United States

## Abstract

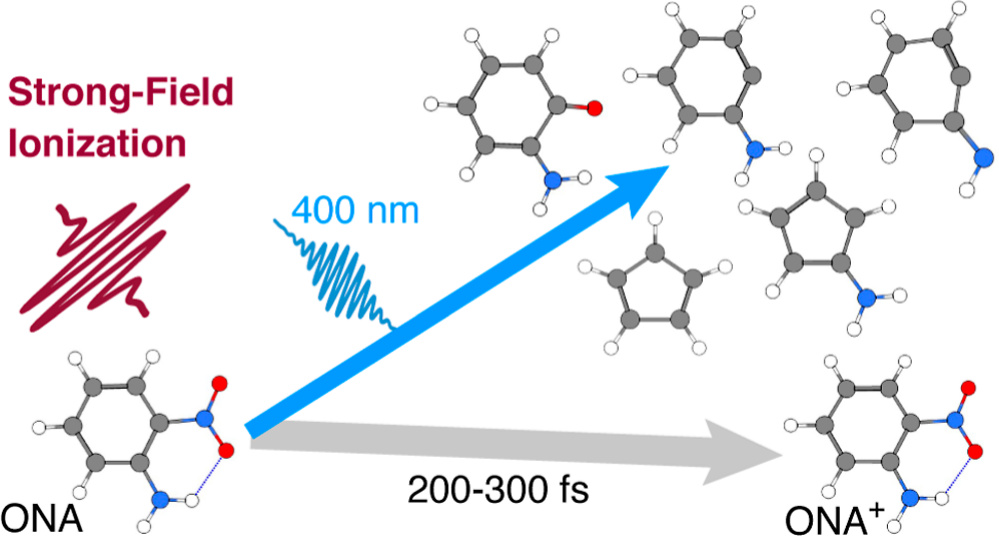

*0rtho*-Nitroaniline (ONA) is a model for the insensitive high explosive 1,3,5-triamino-2,4,6-trinitrobenzene (TATB) that shares strong hydrogen bonding character between adjacent nitro and amino groups. This work reports femtosecond time-resolved mass spectrometry (FTRMS) measurements and theoretical calculations that explain the high stability of the ONA cation compared with related nitroaromatic molecules. Ab initio calculations found that the lowest-lying electronic excited state of the ONA cation, D_1_, lies more than 2 eV above the ground state, and the energetic barriers to rearrangement and dissociation reactions exceed this D_1_ energy. These theoretical results were confirmed by FTRMS pump–probe measurements showing that (1) fragment ions represented less than 30% of the total ion yield when a 10^14^ W cm^–2^, 1300 nm, 20 fs pump pulse was used to ionize ONA; and (2) 3.1 eV (400 nm) photons were required to induce dissociation of the ONA cation. Stronger coupling between the ground D_0_ and excited D_4_ states of the ONA cation at the geometry of neutral ONA resulted in a transient enhancement of fragment ion yields at <300 fs pump–probe delay times, prior to relaxation of the ONA cation to its optimal geometry.

## 1 Introduction

The high explosive 1,3,5-triamino-2,4,6-trinitrobenzene (TATB) is exceptionally insensitive to accidental thermal and shock initiation, making it widely applicable for both defense and civilian purposes.^1^ The unexpected insensitivity of TATB has been attributed to strong hydrogen bonding,^2,3^ π-stacking interactions,^4^ and cooperativity between these phenomena.^5^ In particular, the intramolecular hydrogen bonding between the *ortho* nitro and amino groups in the TATB molecule contributes to its stability to thermal bond dissociation.^6^ Excitation with UV light, in contrast, has long been known to induce photochemical reactions in TATB.^7−10^

**Figure 1:**
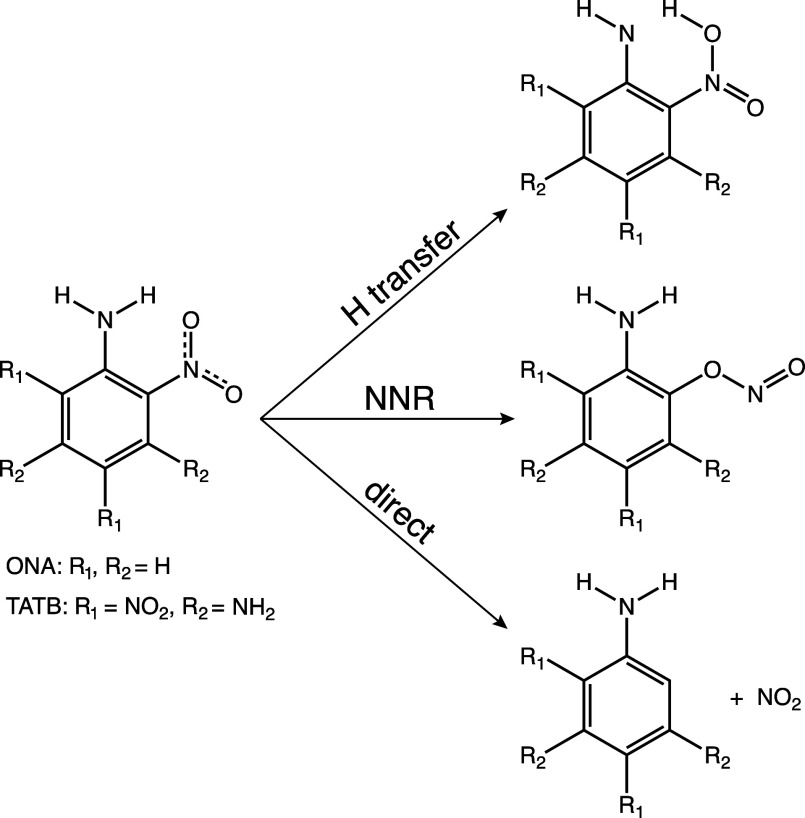
Initial photochemical reaction pathways of ONA and TATB.

*ortho*-Nitroaniline (ONA) can model the photochemistry of TATB because it possesses strong intramolecular hydrogen bonding interactions and undergoes the same three initial reaction pathways as TATB upon UV excitation (Figure 1). First, intramolecular hydrogen transfer from the amino to nitro group in ONA is observed in photoionization mass spectrometry with 266 nm excitation^11^ and matrix-isolation with 185 nm excitation.^12^ In TATB, this pathway produces benzofurazan derivatives by elimination of water from the aci-nitro tautomer.^13,14^ Second, nitro-nitrite rearrangement (NNR) and subsequent NO loss is observed in photoionization mass spectrometry of ONA^11^ and produces the stable TATB radical photoproduct observed with ESR and optical spectroscopy.^9,10^ Finally, direct NO_2_ loss is observed in photoionization mass spectrometry of ONA^11^ and by X-ray photoelectron spectroscopy of TATB.^7^

Shock initiation of energetic materials induces not only electronic excitation but also ionization,^15−17^ making the reactions of ionized energetic molecules important to understanding their initial decomposition pathways. However, despite extensive computational investigations of the reaction pathways in Figure 1 for neutral TATB,^10,13,18−20^ to the best of our knowledge analogous reactions have not been explored in the cations of TATB or ONA. Electron impact ionization produces high yields of the parent cations of both ONA and TATB, unlike the related molecules *ortho*-nitrotoluene and 2,4,6-trinitrotoluene (TNT), which exhibit extensive fragmentation.^21^ Hence, it stands to reason that the strong intramolecular hydrogen bonding between nitro and amino groups that stabilizes neutral TATB also contributes to the stability of TATB and ONA cations.

To gain insight into how adjacent nitro and amino groups stabilize molecular cations, this work presents a combined computational and femtosecond time-resolved mass spectrometry (FTRMS) study of the ONA. FTRMS, also called “ultrafast disruptive probing”,^22^ is a pump–probe method wherein the dynamics of multiple dissociation reactions of molecular cations can be tracked simultaneously on femtosecond–picosecond time scales. Our group has recently reported on the dissociation dynamics of nitrobenzene and nitrotoluene cations with FTRMS, which undergo coherent vibrational excitation upon ionization and readily dissociate upon excitation with low-energy (<2 eV) photons.^23−26^ In the present work, we find that the ONA cation requires excitation with higher-energy (3.1 eV) photons to induce dissociation and exhibits no coherent vibrational dynamics; instead, a transient enhancement of fragmentation is observed in the first ∼300 fs after ionization. The observed dissociation dynamics in the ONA cation are rationalized with detailed calculations of the electronic potential energy surfaces, relaxation dynamics, and reaction pathways.

## 2 Methods

### 2.1 Experimental Section

The full experimental set up has been described in detail previously.^24−27^ Briefly, a commercial Ti/sapphire regenerative amplifier (Astrella, Coherent, Inc.) producing 30 fs, 800 nm, 5 mJ pulses at 1 kHz was used. An optical parametric amplifier (TOPAS Prime) was pumped with 2 mJ of the laser output to produce 20 fs, 1300 nm pulses. The 1300 nm OPA output at an intensity of 10^14^ W cm^–2^ was used as the ionizing “pump” in the pump–probe measurements. Three different “probe” wavelengths at 400, 650, and 800 nm were used; experimental details for generation of each probe wavelength have been described previously.^24−26^ In order to electronically excite the ionized species without creating ions on its own, the probe pulses were kept at a moderate intensity of 8 × 10^12^ W cm^–2^ across all three wavelengths. Peak intensities were determined using previous measurements for the 1300 nm pump,^28^ 800 nm probe,^27^ and 650 nm probe.^25^ The intensity of the 400 nm probe in the FTRMS measurements was estimated based on the ionization threshold of ONA of 1.6 × 10^13^ W cm^–2^ at 1300 nm and assuming the same threshold intensity for ionization at 400 nm based on ADK theory^29^ (Supporting Information, Figure S1). Both the pump and probe pulses were focused with a *f* = 20 cm fused silica lens into the extraction region of our linear time-of-flight (TOF) mass spectrometer with linear polarization of the laser electric field parallel to the TOF axis. ONA was introduced via an effusive inlet into the vacuum chamber under gentle heating to produce a working pressure of 2 × 10^–7^ Torr. Mass spectra were recorded with a 1 GHz digital oscilloscope (LeCroy Waverunner 610Zi); reported ion yields were averaged over 20,000 laser shots.

### 2.2 Theory

Our density functional theory (DFT) calculations of neutral and cationic ONA were conducted using Q-Chem 5.3 software^30,31^ employing the restricted Kohn–Sham formalism for neutral species and the unrestricted formalism for cationic species. Geometry optimizations were performed along with the corresponding frequency calculations to verify the presence of true minima. Then, adiabatic and vertical ionization energies were calculated at different levels of theory and compared to experimental data^21,32^ (Table 1). A comparison between the computed and the experimental quantities shows that the values calculated using the Def2TZVPP basis set^33^ underestimate both ionization energies. Therefore, calculations using this basis set were not further considered.

**Table 1:** Calculated Adiabatic and Vertical Ionization Energies for ONA and Comparison to Experimental Values in Literature

method	IE_ad_ (eV)	IE_vert_ (eV)
B3LYP/6-311+G(d)	8.26	8.39
B3LYP/Def2TZVPP	8.20	8.33
CAM-B3LYP/6-311+G(d)	8.30	8.45
CAM-B3LYP/Def2TZVPP	8.23	8.39
ωB97XD/6-311+G(d)	8.25	8.41
ωB97XD/Def2TZVPP	8.19	8.34
Expt	8.27^a^	8.43^b^

a ref (21).

b ref (32).

Considering only the results obtained using the 6-311+G(d) basis set,^34^ we can see that the three functionals (B3LYP,^35,36^ CAM-B3LYP,^37^ and ωB97XD^38^) estimate the experimental ionization energy within reasonable errors of 0.03 and 0.04 eV for IE_ad_ and IE_vert_, respectively. The long-range corrected CAM-B3LYP functional was chosen over the other two because it was the only one that produced a stable structure of the ONA nitrite cation structure that should be formed by the NNR pathway (Figure 1) on the basis of the C_6_H_6_NO^+^ fragment observed in our FTRMS experiments (see Section 3.2). Therefore, all subsequent DFT calculations were performed at the CAM-B3LYP/6-311+G(d) level.

The possible existence of low-energy conformers for the rearranged species, intermediates, and fragments involved in this work was carefully evaluated. Ground-state conformational ensembles were generated using the conformer-rotamer ensemble sampling tool (CREST) version 2.11.1 developed by the Grimme group.^39−41^ The CREST was developed as a utility and driver program for the semiempirical quantum chemistry package xtb, which was also developed by the Grimme group.^42^ The xtb version employed in this work is version 6.4.1. The CREST uses an iterative metadynamics genetic structure crossing (iMTD-GC) workflow with geometry optimization at the GFN2 level, this latter method falls into the semiempirical extended tight-binding (xTB) family of methods.^43^ The energetic threshold considered for the generation of the conformational ensembles was 1 kcal/mol for most of the species, except for the conformers of the aci-rearranged ONA^+^, for which a threshold of 20 kcal/mol was considered (see further discussion in Section 3.4). Then, the obtained conformers were reoptimized at the CAM-B3LYP/6-311+G(d) level. The lowest energy conformer was considered for further calculations in all cases.

The search for transition states started with the use of the freezing string method^44^ or the relaxed scan of a carefully selected degree of freedom. This produced a guess for the transition state (TS) that was optimized. A frequency calculation of the optimized structure was performed to verify that it possessed only a single imaginary frequency. This guaranteed the finding of a first-order saddle point on the potential energy surface (PES); however, in order to confirm that this TS is the one actually joining the species of interest, an intrinsic reaction coordinate (IRC) calculation^45,46^ was performed.

The energies and oscillator strengths for transitions to electronic excited states in neutral and cationic ONA were performed by equation-of-motion coupled-cluster with single and double (EOM-CCSD) excitations^47,48^ in Q-Chem 5.3 and using time-dependent DFT (TDDFT)^49,50^ as implemented in Gaussian 16.^51^ The TDDFT calculations used the CAM-B3LYP functional with the 6-311+G(d) basis; that same basis set was used for EOM-CCSD calculations. All of these excited state calculations used geometries optimized at the CAM-B3LYP/6-311+G(d) level.

Ab initio molecular dynamics (AIMD), more specifically Born–Oppenheimer molecular dynamics (BOMD), were used in this work at the CAM-B3LYP/6-311G(d) level, as implemented in Q-Chem 5.3. The smaller basis set was employed for computational efficiency. The energies of the optimized ONA cation computed at the CAM-B3LYP/6-311G(d) level agree to within 0.21 eV of the CAM-B3LYP/6-311+G(d) energy. AIMD simulations in the microcanonical (NVE) ensemble with 0.5 fs time steps and 508 fs duration were performed to determine the time required for the relaxation of the ONA cation from the vertical to the optimized geometry. In BOMD calculations, the energies and gradients were calculated at each time step via DFT. The initial nuclear velocities were propagated from frequency calculations to put the zero-point vibrational energy into each normal mode.

## 3 Results and Discussion

This section is organized as follows. First, the electronic states of ONA^+^ at the neutral and cation geometries are reported (Section 3.1) to motivate the series of FTRMS measurements reported in Section 3.2. The observed experimental dissociation dynamics are then rationalized through AIMD simulations (Section 3.3) and reaction pathway calculations (Section 3.4). The full picture of ONA^+^ photodissociation and its relevance to the observed behavior of TATB is then discussed in Section 3.5.

### 3.1 Electronic Structure of ONA and ONA^+^

**Figure 2:**
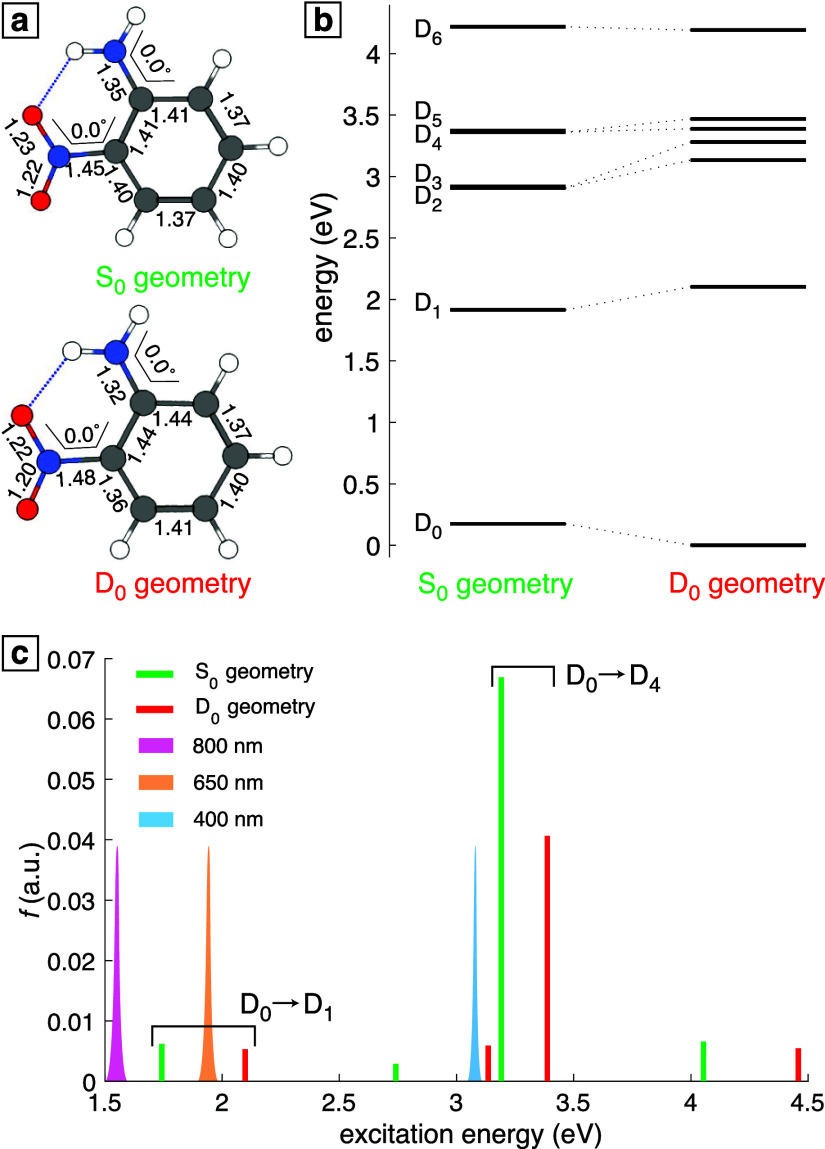
(a) ONA at the S_0_ and D_0_ geometries calculated at the CAM-B3LYP/6-311+G(d) level. (b) Electronic excited states of ONA^+^ at the S_0_ and D_0_ geometries calculated at the EOM-CCSD/6-311+G(d) level (using CAM-B3LYP/6-311+G(d) geometries). (c) Oscillator strengths of transitions out of D_0_ for ONA^+^ at the S_0_ and D_0_ geometries, with approximate spectra of the probe wavelengths in FTRMS. The level of theory is the same as in (b).

Figure 2a shows that both ONA (S_0_ geometry) and ONA^+^ (D_0_ geometry) are planar, with the nitro and amino groups in the plane of the benzene ring (Cartesian coordinates can be found in the Supporting Information, Tables S1 and S2). As seen in the optimized S_0_ and D_0_ structures in Figure 2a and in agreement with literature,^52^ ionization induces only minor changes in the C–N and C–C bond lengths. As a result, the difference between vertical and adiabatic ionization energies is only 0.148 eV, and small shifts in the excited state energies are observed in Figure 2b. The coupling from the ground D_0_ to excited states in the ONA cation (Figure 2c) shows a strong D_0_ → D_4_ transition and weaker D_0_ → D_1_ transition at both S_0_ and D_0_ geometries. For reference, the approximate spectra of the 800, 650, and 400 nm probe pulses are shown to indicate the states that can be populated by excitation with a particular probe wavelength. 800 and 650 nm can access the D_1_ state, whereas 400 nm can access the D_4_ state. We note that the excited state energies of neutral ONA calculated at the TD-CAM-B3LYP level agree with the EOM-CCSD energies to within 0.3 eV and identify the same strongly coupled states; this is particularly true for the low lying excited states (Supporting Information, Table S3). On the other hand, many of the excited states calculated for ONA^+^ at the TD-CAM-B3LYP level presented spin contamination; therefore, these results were not further considered. Tables S4 and S5 contain the excited state energies and oscillator strengths for ONA^+^ at the S_0_ and D_0_ geometries, respectively; only the EOM-CCSD results are shown. Although no calculations of ONA^+^ excited states have been reported to the best of our knowledge, our calculated energy for the neutral ONA S_1_ state of 3.79 eV at the TD-CAM-B3LYP level is reasonably close to literature values for the S_1_ energy of TATB (3.63 eV at the TD-B3LYP/6-311++G(d,p) level)^10^ and ONA (3.06 eV at the same level in water).^53^

The results in Figure 2 show that ONA behaves quite differently from other related nitroaromatic molecules. Previous studies by our group showed that *ortho*-nitrotoluene, a closely related molecule to ONA, went from a C–NO_2_ torsional angle of 26.0 to 35.5° upon ionization.^25^ Ionization was also found to induce changes in the C–NO_2_ torsional angle from 0° to 57.9° in nitrobenzene^23^ and from 0 to 52.5° in *para*-nitrotoluene.^24^ Therefore, an analogous departure from planarity after ionization was expected for ONA. However, the nitro group in the ONA cation remained planar. This behavior suggests a strong intramolecular hydrogen bond between the nitro and amino groups that makes the rotation energetically unfavorable. The electronic structure of the ONA cation also exhibits important differences from the related nitroaromatics. At the S_0_ geometry, both nitrobenzene and *para*-nitrotoluene have at least two excited states below 1 eV.^23,26^ In contrast, the D_1_ state of ONA^+^ at the S_0_ geometry has an energy of approximately 1.74 eV. Moreover, both nitrobenzene and *para*-nitrotoluene have strong coupling to an excited state ∼2 eV above D_0_,^23,26^ but the strongly coupled D_4_ state in ONA is approximately 3.19 eV above D_0_ (at the S_0_ geometry). These differences suggest that ONA will exhibit dissociation dynamics different from those of other nitroaromatic cations, prompting the FTRMS investigations presented below.

### 3.2 FTRMS Measurements

**Figure 3:**
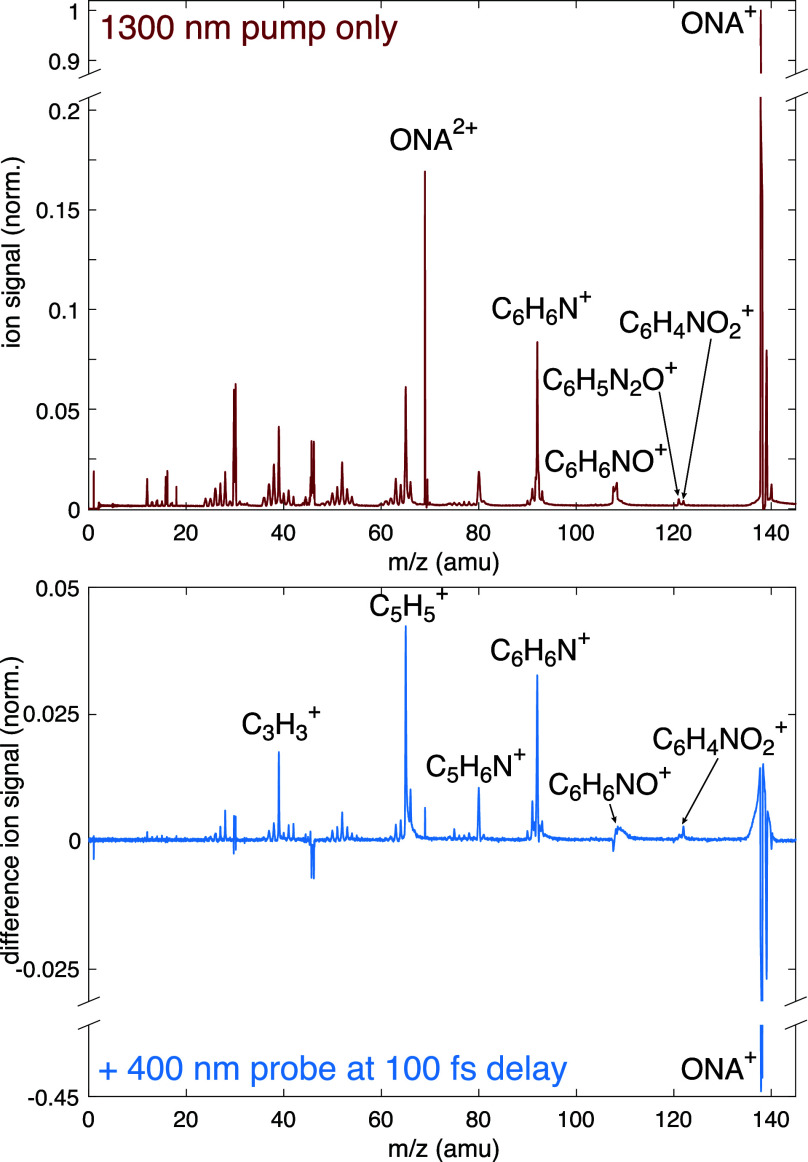
Mass spectrum of ONA taken with 1300 nm pump only (top) and difference spectra relative to the pump only spectrum taken with a 400 nm probe pulse at 100 fs delay (bottom).

Figure 3 shows the mass spectrum of ONA taken with only the 1300 nm pump at an intensity of 10^14^ W cm^–2^ (top) and difference spectrum obtained with addition of the 400 nm probe (8 × 10^12^ W cm^–2^) at 100 fs delay (bottom). To clearly show the low-yield fragment ions, the ordinate axis is broken in both panels; the pump-only spectrum without a broken ordinate axis is shown in the Supporting Information, Figure S2. The most intense signals in the pump-only spectrum were the parent ONA^+^ and dication ONA^2+^; no fragment ion had more than 10% yield relative to the intact parent ion, and the total yield of fragment ions (from ONA^+^, not ONA^2+^) relative to the yield of intact ONA^+^ was 29%. In contrast, the related nitroaromatic cations exhibit significantly higher fragment yields with yields of individual fragment ions being ∼50–100% of the parent ion yield under similar ionization conditions.^23−26^ Moreover, the stability of intact ONA^2+^ contrasts with the *para*-nitrotoluene dication, which undergoes Coulomb explosion to form NO_2_^+^ and C_7_H_7_^+^ within 200 fs.^54^ To our knowledge, no intact dication has previously been reported experimentally in any nitro-organic energetic molecule or model system, although the TATB dication has been considered in DFT calculations.^55^ We note that Coulomb explosion is visible in the NO^+^ and NO_2_^+^ ion signals in Figure 3, arising from dissociation of multiply charged ONA species. ONA, however, is less stable than aniline, which is observed as an intact trication under similar ionization conditions.^56,57^ Hence, the addition of the nitro group to ONA destabilizes the molecule when compared to aniline, but the amino group imparts more stability to the nitro-substituted benzene ring than the methyl group of the nitrotoluenes due to stronger hydrogen bonding.

Of the fragmentation pathways in Figure 1, only the direct pathway to form C_6_H_6_N^+^ (*m*/*z* 92) contributes significantly under pump-only conditions. The NNR and H transfer pathways have a very low probability of occurring because the C_6_H_6_NO^+^ (*m*/*z* 108) and C_6_H_5_N_2_O^+^ (*m*/*z* 121) values are extremely low (Figure 3, top). When the 400 nm probe is added at a 100 fs delay, depletion of ONA^+^ and enhancement of fragment ions are observed (Figure 3, bottom). The greatest enhancement occurs for the direct fragmentation product C_6_H_6_N^+^ and its sequential fragmentation products C_5_H_5_^+^ (*m*/*z* 65) and C_3_H_3_^+^ (*m*/*z* 39). Enhancement of the NNR products C_6_H_6_NO^+^ and C_5_H_6_N^+^ (*m*/*z* 80), as well as the H transfer product C_6_H_5_N^+^ [(*m*/*z* 91), the small peak to the left of C_6_H_6_N^+^] is observed to a smaller degree. The enhancement of these fragmentation pathways suggests that they proceed following absorption of a 400 nm probe photon to promote ONA^+^ from the D_0_ to the D_4_ state, as predicted in Figure 2.

**Figure 4:**
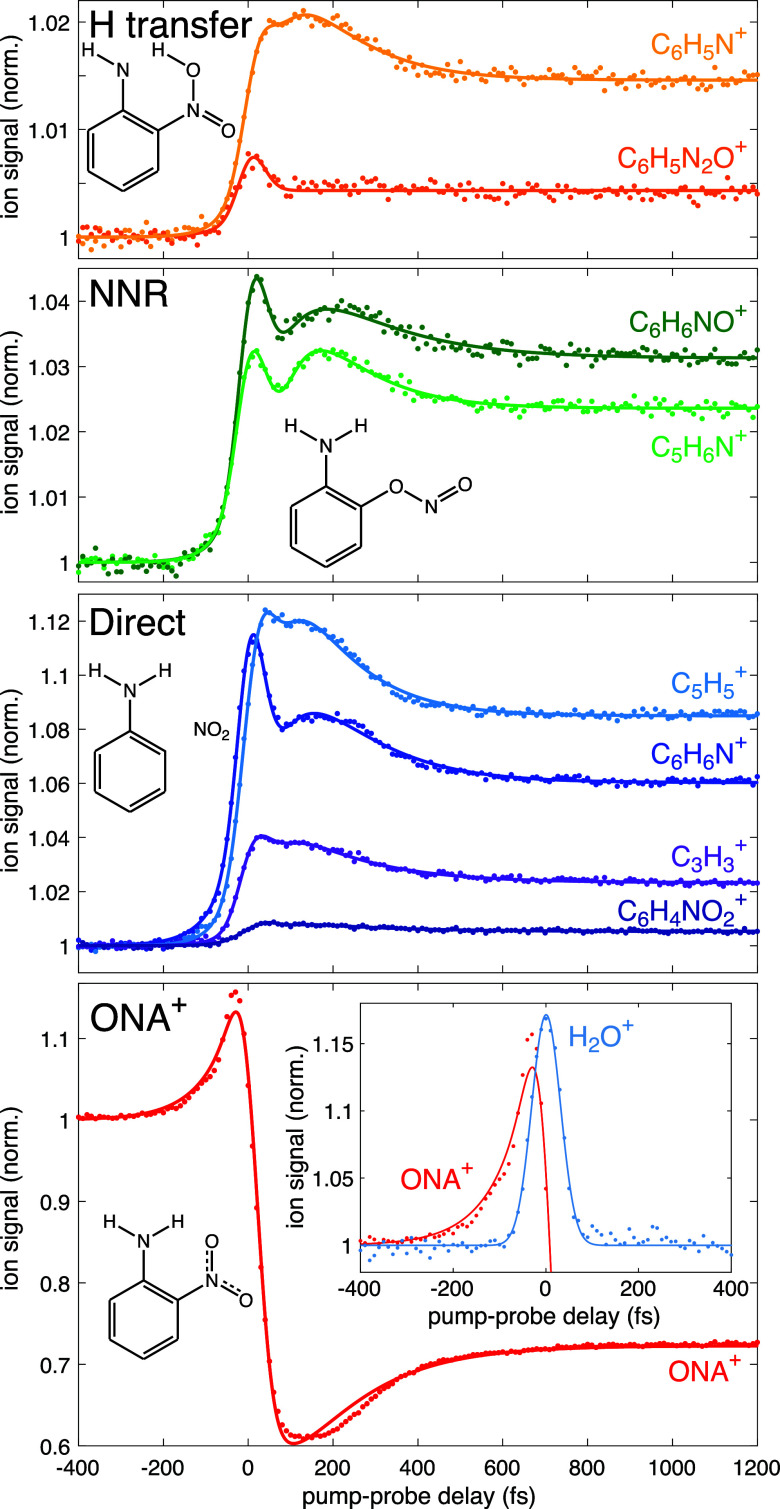
Transient ion dynamics of ONA^+^ and fragments from the direct pathway (blue/violet), NNR pathway (green), and H transfer pathway (yellow/orange). For reference, the molecular structures associated with each pathway are shown. The bottom panel inset shows the enhancement of ONA^+^ compared to the cross-correlation of H_2_O^+^ at negative delays. The dots are data points and solid lines are fits to eq 1.

Figure 4 depicts the FTRMS transient ion signals obtained with 400 nm probing for the species highlighted in Figure 3. Signals were normalized to the ONA^+^ yield and shifted to a value of unity on the ordinate axis at a −400 fs delay in each panel. To ensure that only signals resulting from fragmentation of ONA^+^ (and not ONA^2+^) were recorded, the integration limits were chosen to exclude any signals from Coulomb explosion (side peaks resulting from kinetic energy release following dissociation of a multiply charged cation). Further confirmation that the observed fragment dynamics were due to dissociation of only ONA^+^ was obtained by performing FTRMS measurements with pump intensity of 5 × 10^13^ W cm^–2^, where the yields of signals from Coulomb exploded signals and ONA^2+^ are extremely low. Similar dynamical features as reported in Figure 4 were obtained at this lower pump intensity (Supporting Information, Figure S3). At negative delays (400 nm excitation followed by 1300 nm ionization), the ONA^+^ signal is enhanced relative to the cross-correlation signal from H_2_O^+^ (inset, bottom panel of Figure 4). This enhancement is attributed to excitation of neutral ONA from S_0_ to S_1_, which was observed in earlier literature on TATB^58^ and is consistent with a predicted allowed transition in ONA according to our computations (Supporting Information, Table S3). At positive delays, ONA^+^ undergoes transient depletion for the first 200 fs, followed by partial recovery of the signal over the next 200 fs to reach a constant depleted yield. This transient depletion is mirrored by transient enhancement of the fragment ion signals from each pathway, with the exception of the OH loss product following H transfer, C_6_H_5_N_2_O^+^. The transient for this fragment exhibits a sudden enhancement near zero delay and no further dynamics (Figure 4, top). We note that the transient dynamics in Figure 4 were only observed using 400 nm as the probe wavelength; excitation with 650 or 800 nm resulted in negligible ONA^+^ depletion and fragment ion enhancement (Supporting Information, Figure S4). These results indicate that excitation to D_4_ is necessary to induce dissociation of ONA^+^ because 650 and 800 nm photons can only access the D_1_ state (Figure 2c).

To quantify the transient dynamics in Figure 4, the ion signals were fit using the method introduced by Jochim et al.^22^
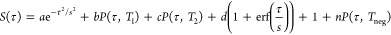
1where τ is the pump–probe delay and *s* = 42.5 fs, which is obtained from the width of the cross-correlation function for H_2_O^+^ seen in the inset of Figure 4. The first term in eq 1 represents the instrument response function from the cross-correlation signal. The terms *P*(τ, *T*_*i*_), *i* = 1, 2 are given by
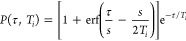
2*T*_1_ and *T*_2_ are associated with dynamics at positive delay (i.e., dynamics corresponding to excitation of the ONA cation for τ > 0). The fourth term in eq 1 accounts for constant depletion or enhancement of the ion signal as τ → ∞ relative to its yield at negative delay. The term *P*(τ, *T*_neg_) is given by
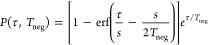
3and represents dynamics at negative delay (i.e., dynamics of ONA in the neutral S_1_ state at τ < 0). All coefficients and time constants in eq 1 are determined by least–squares curve fitting for each transient ion signal shown in Figure 4 and are given in the Supporting Information, Tables S6 and S7.

All ion signals have similar extracted time constants in the ranges of *T*_neg_ ∼ 40–75 fs, *T*_1_ ∼ 30–75 fs, and *T*_2_ ∼ 120–200 fs. The observation of similar *T*_neg_ values for each ion is consistent with the slight enhancement of all signals at a negative delay arising from ionization out of the short-lived S_1_ state. The short *T*_1_ and *T*_2_ values suggest that the dynamics at positive delay may be associated with the relaxation of ONA^+^ from the S_0_ to D_0_ geometry. This claim is further supported by the computational results in Figure 2c indicating that excitation from D_0_ to D_4_ is more efficient at the S_0_ geometry than at the D_0_ geometry. To determine whether the observed transient depletion of ONA^+^ and enhancement of the fragment ions in the first several hundred femtoseconds after ionization is related to geometric relaxation of ONA^+^, we report AIMD simulations below in Section 3.3.

### 3.3 Molecular Dynamics Simulations

Both the experimental observation that ONA^+^ exhibits transient enhancement of fragment ions over the first ∼300 fs after ionization (Figure 4) and the computational result of an appreciable decrease in the oscillator strength for the D_0_ → D_4_ transition when ONA^+^ goes from the S_0_ to D_0_ geometry (Figure 2c) lead to the hypothesis that the observed transient dynamics could be related to the time required for ONA^+^ to relax from the S_0_ to the D_0_ geometry. In order to test this hypothesis, we performed AIMD simulations of this process.

**Figure 5:**
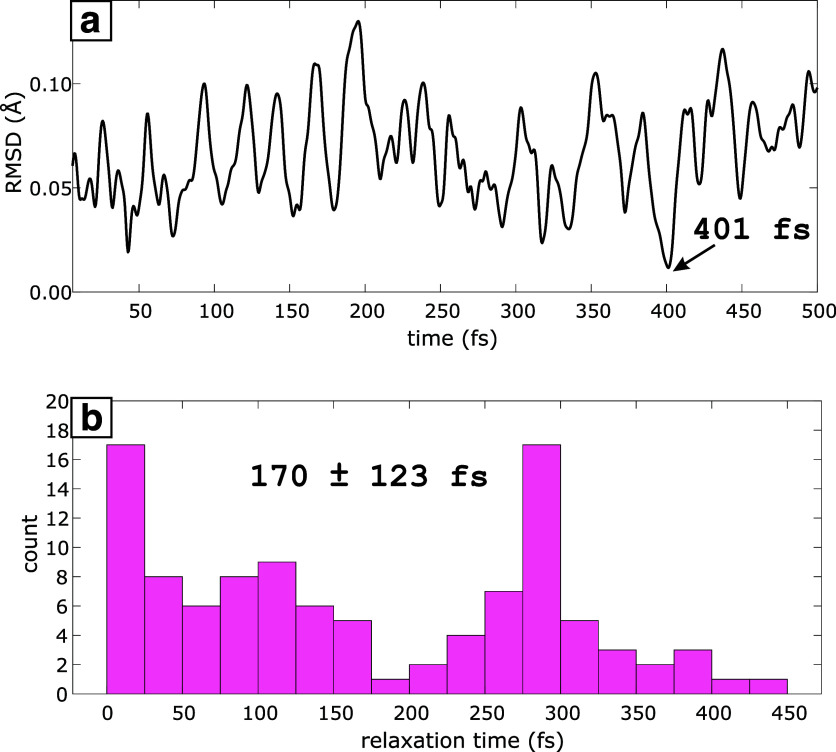
(a) rmsd with respect to ONA^+^ optimized geometry along a sample AIMD trajectory (relaxation time of 401 fs obtained from this trajectory is shown). (b) Histogram for the relaxation times obtained from 105 AIMD trajectories (average and standard deviations shown). Results were obtained at the CAM-B3LYP/6-311G(d) level.

For a given AIMD trajectory, we calculated the root-mean-square deviation (rmsd) for the geometry at every step of the simulation with respect to the optimized ONA D_0_ geometry shown in Figure 2a. Figure 5a shows the evolution of the rmsd along one representative AIMD trajectory out of the 105 trajectories that were calculated. After considering an equilibration time of 6 fs for each simulation, we estimated the relaxation time as the time corresponding to the minimum within each trajectory (401 fs for the particular trajectory shown in Figure 5a).

By performing this procedure along the 105 trajectories, we obtained an average relaxation time of 170 ± 123 fs. Figure 5b shows a histogram of the relaxation times obtained for all of the trajectories. Section SVII of Supporting Information shows an alternative procedure for the estimation of relaxation times based on individual degrees of freedom, such as bond lengths, angles, and dihedrals. This alternative method produced similar relaxation times ranging from 178 ± 123 fs to 233 ± 131 fs, depending on the chosen degree of freedom (Figures S5–S7 and Table S19). The fact that the relaxation times determined from our AIMD simulations are around 170 ± 123 fs follows in line with the hypothesis that the transient depletion of ONA^+^ over the first ∼300 fs after ionization in the FTRMS measurements is related to the time it takes to get from the S_0_ to the D_0_ geometry.

### 3.4 Dissociation Pathways

**Figure 6:**
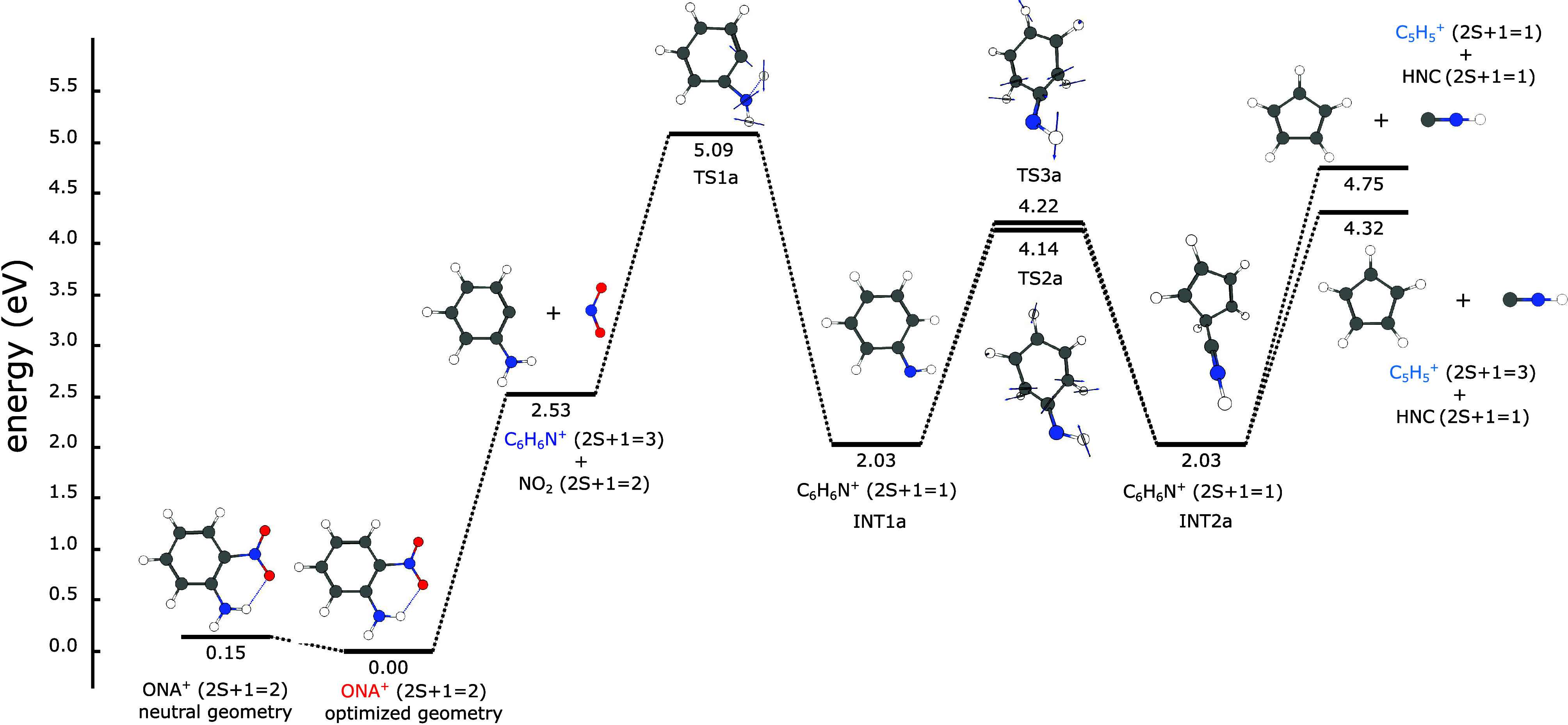
Direct pathway for ONA^+^ fragmentation calculated at the CAM-B3LYP/6-311+G(d) level of theory.

As discussed above, ONA is a good model for TATB because it undergoes the same fragmentation pathways. Although numerous computational investigations of the reaction pathways for neutral TATB have been reported,^10,13,18−20^ there is, to the best of our knowledge, a lack of studies on the reactions of the TATB and ONA cations. This section attempts to fill this gap by presenting a detailed study of the three main fragmentation pathways of the ONA cation. The calculated energies, including the zero-point energy (ZPE), for all the species included in Figures 6 through 9 presented below, as well as the frequencies for all the transition states, can be found in the Supporting Information, Tables S8–S14.

Figure 6 shows the so-called direct pathway for ONA^+^ decomposition, which starts with a barrierless loss of NO_2_ to produce C_6_H_6_N^+^ (*m*/*z* 92), the most intense fragment observed in our experiments (Figure 3). The energy difference between the dissociation products and the reagent of this process is 2.53 eV. The C_6_H_6_N^+^ fragment can then undergo a 1,3-hydrogen shift that needs 2.56 eV to reach the corresponding transition state (TS1a) to generate intermediate INT1a. The ring contraction that occurs between intermediates INT1a and INT2a requires breaking the C–C bond between carbons α and β to the NH group of INT1a. The nonsymmetric position of the H atom in the NH group of INT1a with respect to the two possible β carbons makes it reasonable to assume the existence of two slightly different electronic environments producing two distinct possibilities for the C_α_–C_β_ bond breaking. This situation gives birth to transition states TS2a and TS3a with slightly different geometries and energy. As all the transition states found in this work, TS2a and TS3a were used as a starting point for respective IRC calculations, and a careful analysis of the results revealed that both transition states connect the same intermediates INT1a and INT2a. Then, INT2a undergoes a barrierless process to produce C_5_H_5_^+^ (*m*/*z* 65, observed in experiments) and HNC. There is a long-standing controversy about which of the isomers of C_5_H_5_^+^ is the most stable between the vinylcyclopropenyl singlet cation and the cyclopentadienyl triplet cation with the answer depending on the level of theory used for the computation.^59−61^ These studies agree that the energies of the isomers are close to each other, within 3 kcal/mol (0.13 eV). Due to the nature of the methods employed in this work, the most directly comparable report is the one by Kharnaior and collaborators,^61^ which used the B3LYP/6-311++G(d,p) level of theory among other methods. Their obtained zero point corrected energies showed the cyclopentadienyl triplet cation being more stable by 1.15 kcal/mol, while our calculations at the CAM-B3LYP/6-311+G(d) level showed it to be more stable by 2.05 kcal/mol. Figure 6 shows the generation of the cyclopentadienyl triplet and singlet cations from INT2a, but it should be kept in mind the possibility of also having the vinylcyclopropenyl cation. It is relevant to mention that further fragmentation of C_5_H_5_^+^ has been postulated to produce C_3_H_3_^+^ (*m*/*z* 39),^62,63^ also observed in our experiments. However, due to the plethora of possible C_3_H_3_^+^ formation pathways, including from precursor ions other than C_5_H_5_^+^,^63^ and its lower yield in our experiments, the calculation of C_3_H_3_^+^ formation was deemed beyond the scope of the present work.

**Figure 7:**
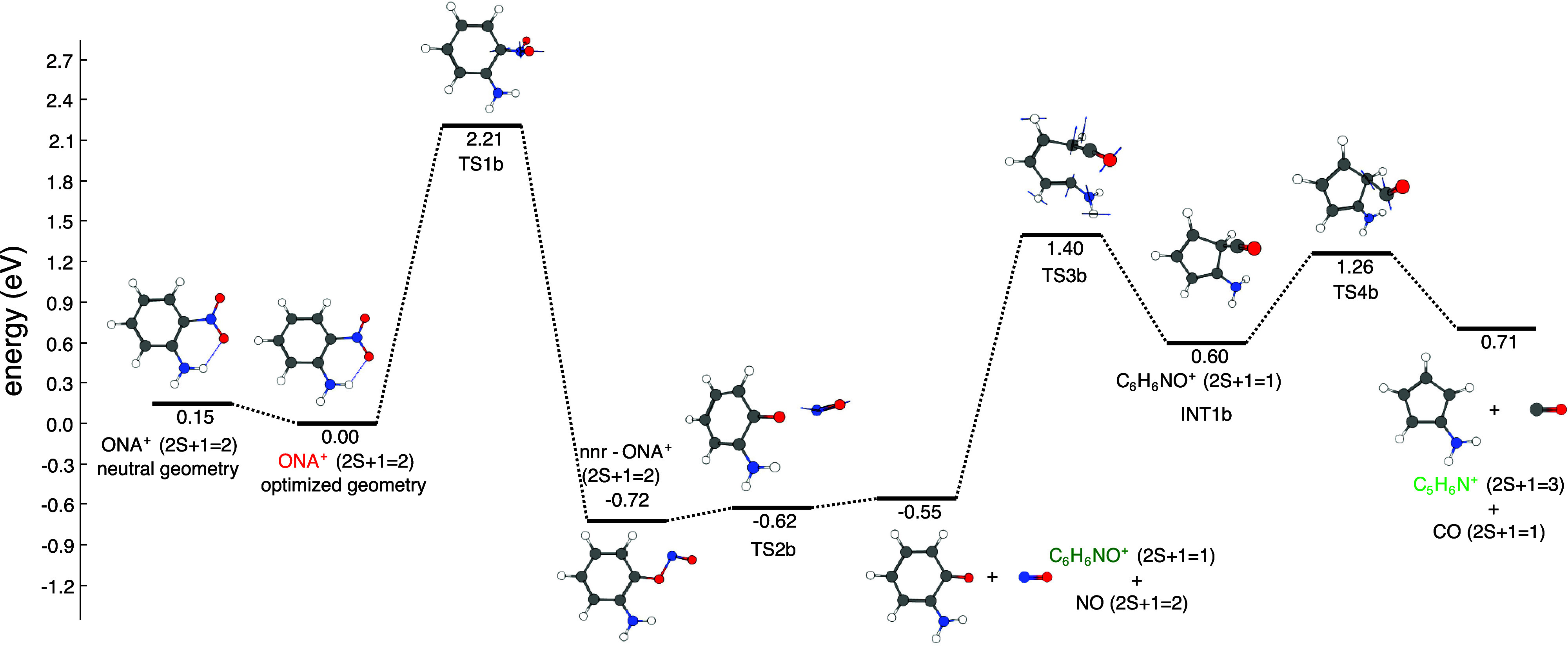
NNR pathway for ONA^+^ fragmentation calculated at the CAM-B3LYP/6-311+G(d) level of theory.

Figure 7 shows the fragmentation pathway that starts with the NNR with a barrier of 2.21 eV associated with transition state TS1b. The nitrite structure, nnr-ONA^+^, dissociates into C_6_H_6_NO^+^ (*m*/*z* 108) and NO through transition state TS2b. It is worth noting the fact that Figure 7 shows TS2b as having an intermediate energy between nnr-ONA^+^ and C_6_H_6_NO^+^ + NO products. This feature arises when localizing minima and transition states on the Born–Oppenheimer surface and then adding ZPE correction.^64^ Considering only electronic energy, the relative energies (with respect to the electronic energy of optimized ONA^+^) of nnr-ONA^+^, TS2b, and C_6_H_6_NO^+^ + NO products are −0.67, −0.44, and −0.51 eV, respectively. Under this scenario, it can be seen that TS2b has higher energy than its corresponding reactant and products. On the other hand, adding the ZPE to all the species, results in TS2b having lower energy than its corresponding products, as shown in Figure 7. Furthermore, the IRC calculation connecting TS2b with reactant and products is done on the Born–Oppenheimer surface, which does not contain a ZPE correction. Overall, this means that from the dynamical point of view, even at 0 K, the conversion of nnr-ONA^+^ to C_6_H_6_NO^+^ + NO products can be considered as barrierless. The detection of C_6_H_6_NO^+^ in our experiments can be deemed as a confirmation that ONA^+^ undergoes NNR. We note that the C_6_H_6_NO^+^ peak in the mass spectrum reported in the bottom panel of Figure 3 is quite broad, suggesting that the nnr-ONA^+^ structure is metastable and that the dissociation step through TS2b occurs over multiple nanoseconds. C_6_H_6_NO^+^ can undergo a ring contraction to generate intermediate INT1b after surpassing a barrier of 1.95 eV associated with the transition state TS3b. Finally, INT1b dissociates into C_5_H_6_N^+^ and CO fragments, with the former being detected as the *m*/*z* 80 fragment in our experiments. It is notable that once ONA^+^ surpasses the initial NNR transition state (TS1b), it has sufficient energy to form both C_6_H_6_NO^+^ and C_5_H_6_N^+^.

**Figure 8:**
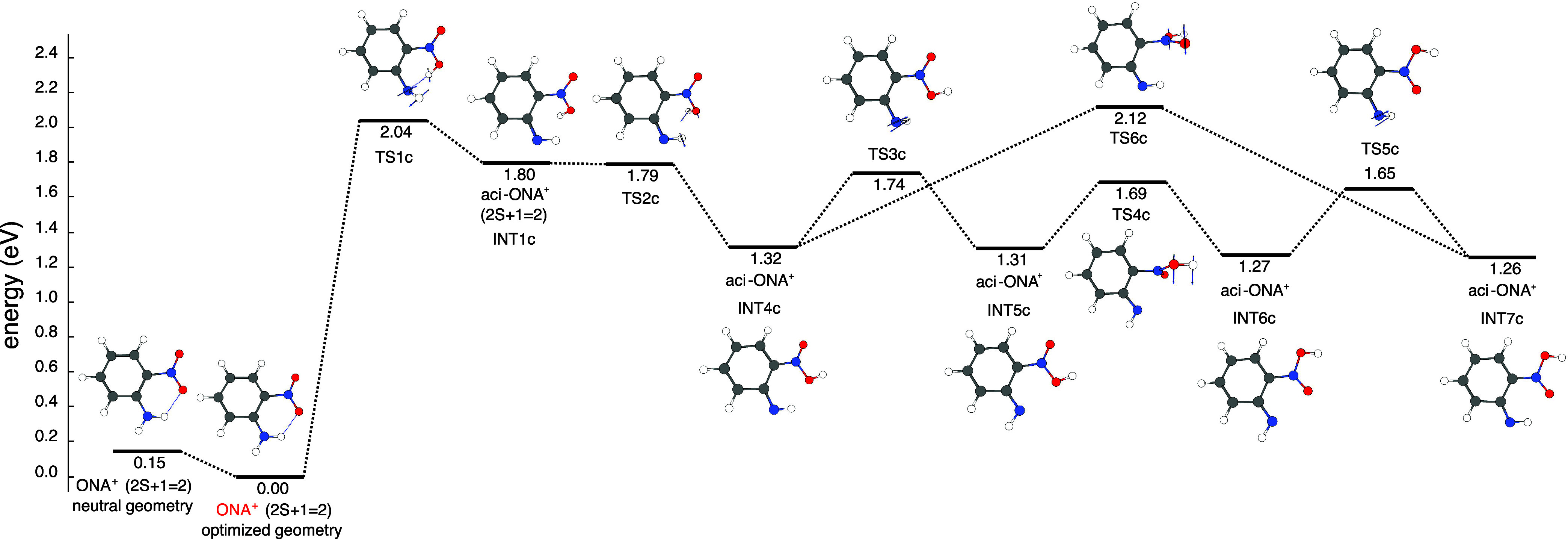
First part of the H transfer pathway for ONA^+^ fragmentation calculated at the CAM-B3LYP/6-311+G(d) level of theory.

Figure 8 shows the first part of the aci-rearrangement pathway of ONA^+^. At this point, it is worth discussing the methodology employed for obtaining the intermediates INT1c and INT4c to INT7c shown in this pathway. A previous study from our group exploring the aci-rearrangement pathway of the *ortho*-nitrotoluene cation (ONT^+^)^25^ found three different conformers of the aci-rearranged ONT^+^ within a window of approximately 12 kcal/mol. As mentioned in Section 2.2, CREST/xtb was used to explore the conformational landscape of the different species considered in this work; in view of the previous results for the ONT^+^ complex, a wide threshold of 20 kcal/mol for the exploration of the conformers of the aci-rearranged ONA^+^ was chosen. From this methodology, seven different conformers within an energetic window of 12.5 kcal/mol (0.54 eV) were found, four of which participate in the pathway shown in Figure 8. Cartesian coordinates for the seven conformers obtained, from INT1c to INT7c, can be found in the Supporting Information, Tables S15–S18. The pathway starts with hydrogen transfer between the amino and the nitro group of ONA^+^ to form transition state TS1c, which lies 2.04 eV above relaxed ONA^+^. This leads to the intermediate INT1c, which, then, through the transition state TS2c, converts to INT4c. The pathway can then proceed in two different ways toward INT7c: it can go in a stepwise fashion through intermediates INT5c and INT6c with energetic barriers around 0.4 eV or it can proceed directly to INT7c with a barrier of 0.8 eV. The existence of all these intermediates and rearrangements between them seem to be enabled by the hydrogen-bonding interactions that can be formed in multiple ways.

**Figure 9:**
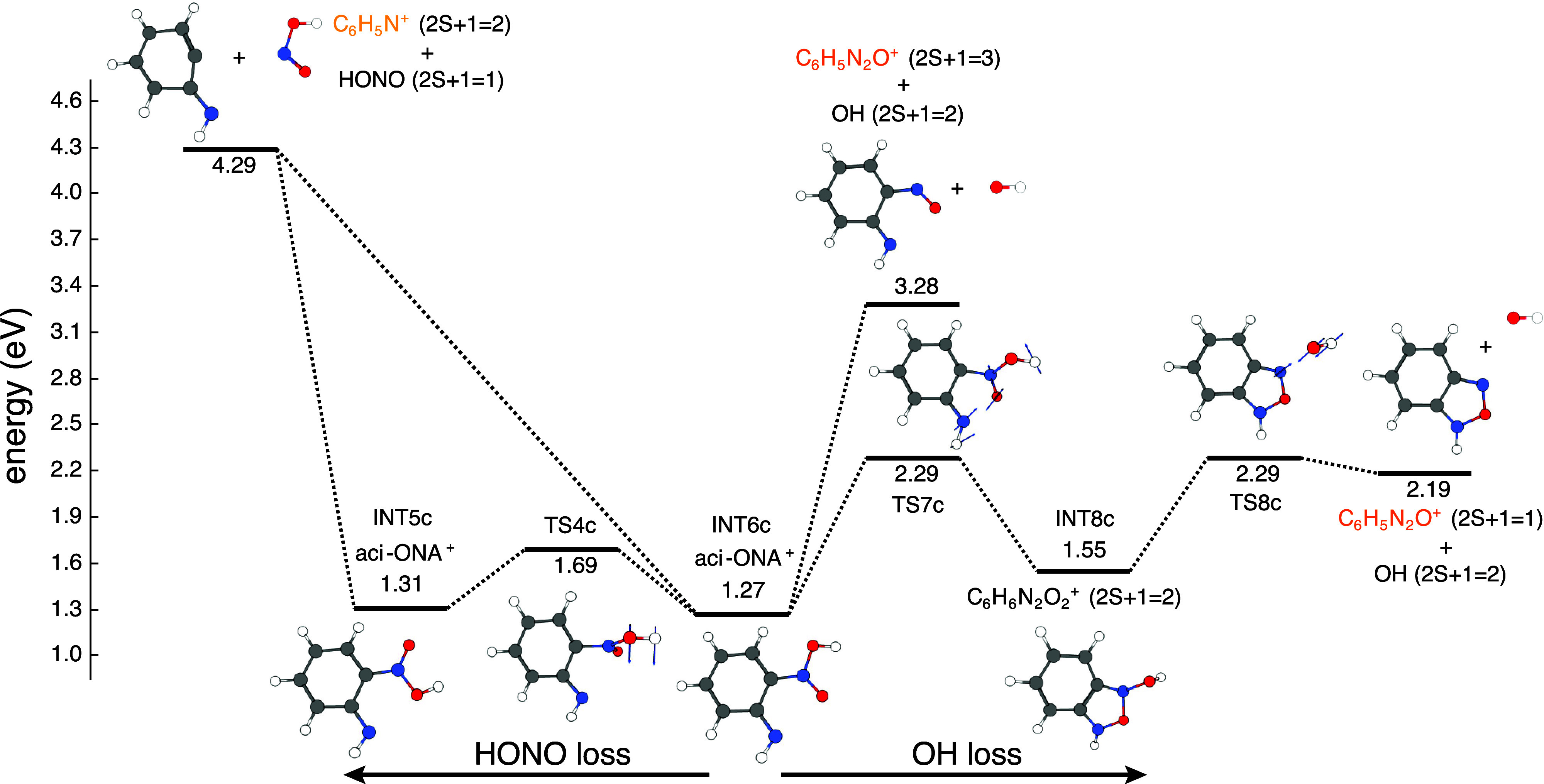
Second part of the H transfer pathway for ONA^+^ fragmentation calculated at the CAM-B3LYP/6-311+G(d) level of theory.

These aci-rearrangement pathways have not yet generated any fragment from ONA^+^. Figure 9 shows the fragmentation steps starting from intermediates INT5c and INT6c in Figure 8. Both INT5c and INT6c can undergo the direct loss of nitrous acid (HONO) to form experimentally detected C_6_H_5_N^+^ (*m*/*z* 91). From both INT5c and INT6c, the dissociation requires approximately 3 eV. In contrast to HONO loss, OH loss was found to only proceed from INT6c. In this regard, there are two possibilities: it can proceed directly with the generation of C_6_H_5_N_2_O^+^ in the triplet state, with an energy difference of 2.01 eV or it can go through the generation of a cyclic intermediate (INT8c) to form C_6_H_5_N_2_O^+^ in the singlet state.

### 3.5 Discussion

**Figure 10:**
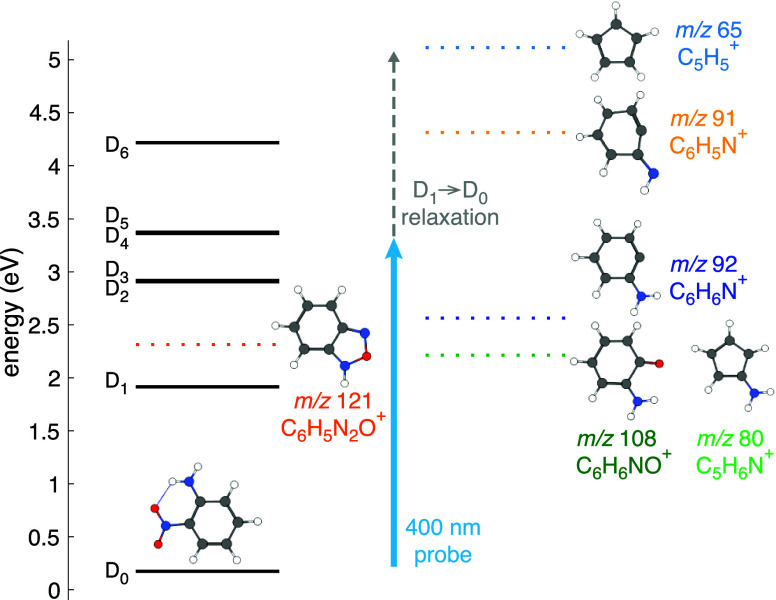
Summary of reaction pathways for ONA^+^.

The experimental and computational results presented above paint a complex picture of photodissociation pathways in the ONA cation. Figure 10 summarizes these results using the computed energy levels of the ONA cation at S_0_ geometry (black solid lines; cf., Section 3.1) and energetic barriers to formation of the experimentally observed fragment ions (dotted colored lines, cf., Section 3.4). The first feature to point out is that the D_1_ state lies at least 0.3 eV below all of the dissociation barriers. Hence, any ONA molecules that are ionized into D_1_ by the pump pulse in our experiments are expected to relax to the D_0_ state without dissociating. Due to nonadiabatic coupling between electronic states, electronic relaxation to D_0_ would be expected to proceed within ∼50 fs of ionization, as recently observed in the naphthalene cation.^65^ Hence, all molecules can be assumed to be in the D_0_ state by the end of the ∼70 fs fwhm instrument response function, when the 400 nm probe pulse (blue arrow in Figure 10) interacts with ONA^+^. Some of these ONA^+^ molecules are expected to have excess vibrational energy gained from D_1_ → D_0_ relaxation, as shown by the dashed gray line in Figure 10. Absorption of a probe photon promotes ONA^+^ to D_4_, giving it enough energy to dissociate into C_6_H_6_N^+^, C_6_H_6_NO^+^, and C_5_H_6_N^+^. The population of vibrationally excited ONA^+^ molecules could further dissociate into C_6_H_5_N^+^ and C_5_H_5_^+^ with excess energy gained from relaxation from D_1_ → D_0_.

The fragment ions C_6_H_6_N^+^, C_6_H_6_NO^+^, C_5_H_6_N^+^, C_6_H_5_N^+^, and C_5_H_5_^+^ exhibited transient enhancement over the first ∼300 fs after ionization, consistent with the time required for ONA^+^ to relax from the S_0_ to D_0_ geometry (Section 3.3). The transient enhancement is due to ONA^+^ under S_0_ geometry having a considerably higher oscillator strength and better overlap with the 400 nm probe spectrum compared with the D_0_ geometry (Figure 2c). This increased probability of exciting the cation to its D_4_ state prior to geometric relaxation results in a higher yield of dissociation products resulting from three main pathways: direct dissociation, NNR, and H-transfer.

Besides the three main fragmentation pathways extensively discussed in the present work, we have evidence of the existence of an additional direct pathway leading to the generation of C_6_H_4_NO_2_^+^ (Figure 4). This fragment is generated by the barrierless loss of NH_2_ as confirmed computationally through a relaxed scan of the C–NH_2_ bond. The energy difference between products and reactants for this process is 5.78 eV, which is a value higher than the energetic barriers shown in Figure 10. The experimental presence of this fragment can be rationalized by an argument analogous to the one previously exposed for the generation of C_6_H_5_N^+^ and C_5_H_5_^+^: it might be possible to have some ONA molecules being ionized into D_2_ or even higher excited states, and once they relax to the D_0_ state, they could constitute a population with excess vibrational energy amenable of being further excited with the 400 nm probe photons, gaining enough energy for the NH_2_ loss.

In contrast with the above-mentioned dissociation products, fragment C_6_H_5_N_2_O^+^ (*m*/*z* 121) does not exhibit enhanced yield at short pump–probe delays prior to ONA^+^ relaxation, as seen in the top panel of Figure 4. It is possible that the low yield and noisy signal of this ion mask such dynamics in our experiment. Alternatively, this result could indicate that OH loss through the H-transfer pathway is not induced by a D_0_ → D_4_ transition with a 400 nm probe pulse. Instead, the small yield of C_6_H_5_N_2_O^+^ in our mass spectra could come from ONA molecules that are ionized into the nearly degenerate D_2_/D_3_ states and rapidly undergo H atom transfer. Previous computational results from our group found that H-transfer in the related *ortho*-nitrotoluene cation can proceed within 20 fs.^25^ Hence, one may expect that electronically excited ONA^+^ formed by the pump pulse would complete the initial H-transfer to form the aci-ONA^+^ structure(s) within the time of the instrument response. As a result, the 400 nm probe pulse would interact with aci-ONA^+^, which could have significant changes in its excited states compared to ONA^+^ such that no allowed transition is available. Because HONO loss from the H-transfer pathway to produce C_6_H_5_N^+^ (*m*/*z* 91) does exhibit transient enhancement, we can conclude that it does form from the D_0_ → D_4_ transition, with excess energy from initial electronic relaxation, as described above.

The observation that the majority of fragment ions from ONA^+^ are most easily formed during the short ∼200–300 fs period during which ONA^+^ undergoes geometric relaxation can explain the widely observed stability of ONA^+^ under electron-impact ionization.^21^ The stability of ONA^+^ is further confirmed by our electronic structure and reaction pathway computations. In particular, it is notable that the energetic barriers obtained in this work for the fragmentation of ONA^+^ are quite similar to the analogous reaction pathways in neutral TATB obtained using DFT methods^13,18,20^ (Table 2). Hence, the insights into the stability of the ONA cation gained in this work are of direct relevance to understanding the initial decomposition behavior of TATB.

**Table 2:** Calculated Energetic Barriers to Fragment Formation in ONA^+^ from This Work and Neutral TATB from Literature as Indicated

reaction	ONA^+^ (eV)	TATB (eV)
direct NO_2_ loss	2.53	2.97;^18^ 3.25;^13^ 3.16^20^
NNR and NO loss	2.21	2.38;^13^ 2.32^20^
H-transfer and OH loss	2.29	2.84;^13^ 2.64^20^
H-transfer and HONO loss	4.29	4.13;^13^ 3.52^20^

## 4 Conclusions

This work offers some insights on the dynamics, stability, and fragmentation pathways of the ONA radical cation as a model for the military explosive TATB. Electronic structure calculations showed a strong D_0_ to D_4_ transition with an energy of 3.4 eV capable of inducing fragmentation, as confirmed by FTRMS measurements. This transition requires a higher energy than the bright transition at ∼2 eV that induces fragmentation in related nitroaromatic radical cations previously studied,^23−26^ giving evidence of the greater stability of ONA^+^. The oscillator strength and therefore the intensity of the D_0_ to D_4_ transition is reduced as the ONA cation relaxes from the S_0_ to D_0_ geometry, which according to our AIMD simulations requires approximately 200–300 fs. These results explain the transient enhanced fragmentation of ONA^+^ observed during the first 300 fs of our FTRMS measurements. Finally, we present detailed mechanisms for the direct, NNR, and H-transfer dissociation pathways in ONA^+^, which have similar energetic barriers to the analogous reaction pathways in neutral TATB. These results on ONA^+^ motivate future investigations into the properties of the TATB cation to investigate whether it exhibits similar stability that can contribute to TATB’s insensitivity as a high explosive.

## Supporting Information Available

Additional theoretical results including molecular geometries, energy levels, fit coefficients, oscillator strengths, and frequencies; additional data for FTRMS measurements; and histograms of AIMD trajectory results (PDF)
