# Supplementary material for: Photodissociation Dynamics of the Highly Stable ortho-Nitroaniline Cation
Source: J Phys Chem A. 2024 Feb 27;128(9):1634–45. doi: 10.1021/acs.jpca.3c08364 (PMC10926099; doi:10.1021/acs.jpca.3c08364)
Supplement: Supplementary file 1 — jp3c08364_si_001.pdf [file jp3c08364_si_001.pdf]

Supporting Information: Photodissociation Dynamics of the Highly Stable *ortho*-Nitroaniline Cation

Hugo A. López Peña, Jacob M. Shusterman, Clayton Dalkiewicz, Shane L. McPherson, Christine Dunstan, Kunjal Sangroula, Ka Un Lao, and Katharine Moore Tibbetts*

Department of Chemistry, Virginia Commonwealth University, Richmond, VA 23284, United States

E-mail: kmtibbetts@vcu.edu

Contents

Number of pages: 19

Number of figures: 8

Number of tables: 19

SI Geometries and Excited States of ONA Neutral and Cation

Table S1: Cartesian coordinates for neutral ONA optimized at the CAM-B3LYP/6-311+G* level.

Element	X	Y	Z
C	0.4319819470	-0.9926778540	0.0000002497
C	-0.2254560770	0.2571687080	-0.0000011288
C	1.8441320740	-0.9525065940	0.0000016860
C	2.5358322520	0.2321727150	0.0000004262
C	0.4906608880	1.4584852240	-0.0000027345
C	1.8628811540	1.4594119350	-0.0000013873
N	-0.1903660160	-2.1948085170	0.0000003827
H	0.3553894100	-3.0377550110	0.0000074911
H	-1.1944469480	-2.2453795690	-0.0000025625
N	-1.6725795900	0.3582043580	0.0000001972
O	-2.3327492930	-0.6789305760	-0.0000046052
O	-2.1798416000	1.4637265840	0.0000057649
H	2.3814637960	-1.8951253170	0.0000036078
H	3.6200760000	0.2100407220	0.0000018873
H	2.4111488630	2.3926613210	-0.0000032067
H	-0.0724781690	2.3810941010	-0.0000032211

Table S2: Cartesian coordinates for ONA cation optimized at the CAM-B3LYP/6-311+G* level.

Element	X	Y	Z
C	0.4694221212	-0.9778555500	0.0000000000
C	-0.2450409797	0.2737410798	0.0000000000
C	1.9042624592	-0.9254495831	0.0000000000
C	2.5668361022	0.2712078023	0.0000000000
C	0.4317530036	1.4546813938	0.0000000000
C	1.8425962568	1.4691716441	0.0000000000
N	-0.1246052099	-2.1545361318	0.0000000000
H	0.4154651499	-3.0115025229	0.0000000000
H	-1.1430444257	-2.2043045977	0.0000000000
N	-1.7250829616	0.3281269338	0.0000000000
O	-2.3213302049	-0.7389482214	0.0000000000
O	-2.2381721704	1.4130744390	0.0000000000
H	2.4507701463	-1.8621450423	0.0000000000
H	3.6494803843	0.2912748471	0.0000000000
H	2.3602020593	2.4206237412	0.0000000000
H	-0.1303183871	2.3801893688	0.0000000000

Table S3: Excited state energies and oscillator strengths for neutral ONA.

	TDDFT/CAM-B3LYP/6-311+G*		EOM-CCSD/6-311+G*	
State	Energy	f	Energy	f
S1	3.7898	0.1328	3.9996	0.1487
S2	4.0879	0.0000	4.1609	0.0000
S3	4.6583	0.0005	4.7875	0.0006
S4	5.0847	0.0772	5.3369	0.0394
S5	5.6075	0.1676	5.4933	0.0049
S6	5.6486	0.0035	5.9502	0.2905
S7	5.9712	0.1385	6.1491	0.0168
S8	6.2864	0.0002	6.1870	0.0003
S9	6.4233	0.1744	6.3620	0.0061
S10	6.4990	0.0090	6.3792	0.3810

Table S4: Excited state energies and oscillator strengths for ONA cation at the S_0 geometry.

	EOM-CCSD/6-311+G*	
State	Energy	f
D1	1.7427	0.0062
D2	2.7408	0.0029
D3	2.7497	0.0000
D4	3.1908	0.0669
D5	3.2063	0.0000
D6	4.0537	0.0066

Table S5: Excited state energies and oscillator strengths for ONA cation at the D_0 geometry.

	EOM-CCSD/6-311+G*	
State	Energy	f
D1	2.0980	0.0053
D2	3.1354	0.0059
D3	3.2846	0.0000
D4	3.3871	0.0406
D5	3.4654	0.0000
D6	4.1955	0.0000

SII Fit Coefficients

Reproduce the equation.

$$S(\tau) = ae^{-\tau^2/s^2} + bP(\tau, T_1) + cP(\tau, T_2) + d(1 + \text{erf}\left(\frac{\tau}{s}\right)) + 1 + nP(\tau, T_{\text{neg}}) \tag{S.1}$$

Table S6: Coefficients extracted from curve fitting for direct dissociation pathways.

Coeff.	ONA^+	$C_6H_6N^+$	$C_5H_5^+$	$C_3H_3^+$	$C_6H_4NO_2^+$
a	0.09±0.03	0.042±0.004	0.021±0.009	0.014±0.006	0.003±0.003
b	0.19±0.11	-0.11±0.06	-0.15±0.04	-0.04±0.03	-0.02±0.02
T_1	67±40	75±29	49±11	41±16	32±12
c	-0.19±0.15	0.08±0.07	0.08±0.02	0.018±0.004	0.003±0.001
T_2	159±45	144±39	130±15	184±25	208±75
d	-0.139±0.001	0.0301±0.0002	0.0425±0.0001	0.0116±0.0001	0.0026±0.0001
n	0.11±0.02	0.034±0.005	0.029±0.005	0.008±0.003	0.004±0.001
T_{neg}	75±10	55±5	42±4	43±10	37±7

Table S7: Coefficients extracted from curve fitting for NNR and H transfer pathways.

Coeff.	$C_6H_6NO^+$	$C_5H_6N^+$	$C_6H_5N_2O^+$	$C_6H_5N^+$
a	0.016±0.002	0.016±0.002	0.003±0.002	0.002±0.002
b	-0.03±0.01	-0.07±0.02		-0.02±0.01
T_1	72±52	59±23		62±50
c	0.02±0.02	0.03±0.03		0.02±0.02
T_2	184±90	124±37		133±58
d	0.0156±0.0002	0.0118±0.0001	0.0021±0.0001	0.0072±0.0001
n	0.010±0.003	0.013±0.003	0.0014±0.0009	0.004±0.002
T_{neg}	46±9	42±5	57±12	45±14

SIII Pathway Energies

Table S8: Direct pathway energies (E) calculated at the CAM-B3LYP/6-311+G* level. Energies include ZPE.

Species	Calc. E (Hartree)	E within pathway (Hartree)	Rel. E (eV)
ONA+ (S0)	-491.5832235	-491.5832235	0.15
ONA+ (D0)	-491.5886553	-491.5886553	0
C6H6N+	-286.4344729	-	-
NO2	-205.0612241	-	-
C6H6N+ + NO2	-491.495697	-491.495697	2.53
TS1a	-286.3405118	-491.4017359	5.09
INT1a	-286.4529953	-491.5142194	2.03
TS2a	-286.3752523	-491.4364764	4.14
TS3a	-286.3721775	-491.4334015	4.22
INT2a	-286.453014	-491.5142381	2.03
C5H5+ (triplet)	-193.0030022	-	-
HNC	-93.36579443	-	-
C5H5+ (triplet) + HNC	-286.3687966	-491.4300207	4.32
C5H5+ (singlet)	-192.9871507	-	-
C5H5+ (singlet) + HNC	-286.3529451	-491.4141692	4.75

Table S9: NNR pathway energies (E) calculated at the CAM-B3LYP/6-311+G* level. Energies include ZPE.

Species	Calc. E (Hartree)	E within pathway (Hartree)	Rel. E (eV)
ONA+ (S0)	-491.5832235	-491.5832235	0.15
ONA+ (D0)	-491.5886553	-491.5886553	0
TS1b	-491.5075266	-491.5075266	2.21
nnr-ONA+	-491.6151207	-491.6151207	-0.72
TS2b	-491.6113184	-491.6113184	-0.62
C6H6NO+	-361.7263474	-	-
NO	-129.8825705	-	-
C6H6NO+ + NO	-491.6089179	-491.6089179	-0.55
TS3b	-361.6544785	-491.537049	1.4
INT1b	-361.6841108	-491.5666813	0.6
TS4b	-361.6599329	-491.5425034	1.26
C5H6N+	-248.3794051	-	-
CO	-113.3004769	-	-
C5H6N+ + CO	-361.679882	-491.5624525	0.71

Table S10: H transfer pathway energies (E) calculated at the CAM-B3LYP/6-311+G* level. Energies include ZPE.

Species	Calc. E (Hartree)	E within pathway (Hartree)	Rel. E (eV)
ONA+ (S0)	-491.5832235318	-491.5832235318	0.15
ONA+ (D0)	-491.5886553450	-491.5886553450	0.00
TS1c	-491.5137557821	-491.5137557821	2.04
INT1c	-491.5224392374	-491.5224392374	1.80
TS2c	-491.5229063428	-491.5229063428	1.79
INT4c	-491.5399743033	-491.5399743033	1.32
TS3c	-491.5246173211	-491.5246173211	1.74
INT5c	-491.5405814143	-491.5405814143	1.31
TS4c	-491.5265602425	-491.5265602425	1.69
INT6c	-491.5421659724	-491.5421659724	1.27
TS5c	-491.5280257237	-491.5280257237	1.65
INT7c	-491.5424793381	-491.5424793381	1.26
TS6c	-491.5108858807	-491.5108858807	2.12
C6H5N+	-285.7580626320	-	-
HONO	-205.6729629576	-	-
C6H5N+ + HONO	-491.4310255896	-491.4310255896	4.29
TS7c	-491.5044261129	-491.5044261129	2.29
INT8c	-491.5315175234	-491.5315175234	1.55
TS8c	-491.5045906773	-491.5045906773	2.29
C6H5N2O+ (singlet)	-415.7880930450	-	-
OH	-75.7202580406	-	-
C6H5N2O+ (singlet) + OH	-491.5083510857	-491.5083510857	2.19
C6H5N2O+ (triplet)	-415.7479171581	-	-
C6H5N2O+ (triplet) + OH	-491.4681751987	-491.4681751987	3.28

SIV Vibrational Frequencies of Transition States

Table S11: Harmonic frequencies (cm^{-1}) and intensities (km mol^{-1}) for transition states within direct pathway, calculated at the CAM-B3LYP/6-311+G* level.

TS1a		TS2a		TS3a	
Frequency	Intensity	Frequency	Intensity	Frequency	Intensity
-2319.05	138.305	-699.13	112.391	-740.41	132.225
203.81	4.186	166.08	0.53	168.01	0.323
287.73	156.851	276.46	6.989	264.98	61.365
383.46	9.997	432.84	0.855	428.45	1.882
445.1	4.59	460.67	8.661	469.79	24.063
468.81	8.267	521.97	51.283	492.28	88.414
553.28	1.673	593.91	12.098	581.8	18.498
627.2	5.531	631.78	9.847	605.06	15.011
662.02	17.206	706.25	74.244	707.27	149.336
780.79	61.686	745.33	9.527	730.52	14.581
868.79	6.609	858.22	32.275	828.08	22.08
879.27	0.296	909.01	68.356	885.65	20.648
922.78	21.444	950.17	33.128	950.44	4.601
947.73	35.388	979.75	13.297	970.35	9.902
984.96	0.465	990.24	14.617	986.55	2.74
989.23	6.427	1000.01	2.131	1002.22	2.066
1035.13	0.253	1009.8	6.058	1011.96	6.683
1041.73	39.083	1074.98	224.177	1060.27	35.59
1135.92	6.702	1090.17	116.525	1102.4	103.414
1191.03	4.484	1155.82	5.165	1154.54	4.201
1270.32	12.405	1188.56	53.203	1174.71	2.904
1334.17	9.866	1304.2	11.405	1297.63	21.221
1407.76	10.906	1379.3	8.905	1366.05	8.782
1457.88	20.629	1399.7	34.093	1399.63	37.128
1524.92	10.593	1642.19	5.81	1645.88	9.241
1574.49	17.75	1671.84	5.599	1677.58	3.178
1654.65	91.473	1875.68	59.428	1858.82	117.159
1897.12	31.73	3149.98	25.788	3143.42	24.269
3221.74	0.794	3200.69	50.231	3201.06	49.556
3233.8	2.098	3210.43	12.942	3213.52	24.064
3247.88	6.111	3227.25	20.876	3229.14	35.367
3258.92	10.933	3255.56	6.714	3254.98	6.184
3542.69	310.027	3521.84	73.916	3521.36	106.138

Table S12: Harmonic frequencies (cm^{-1}) and intensities (km mol^{-1}) for transition states within NNR pathway, calculated at the CAM-B3LYP/6-311+G* level.

TS1b		TS2b		TS3b		TS4b	
Frequency	Intensity	Frequency	Intensity	Frequency	Intensity	Frequency	Intensity
-935.28	405.825	-92.13	279.277	-208.87	83.347	-573.53	213.36
103.09	2.521	53.97	1.593	96.44	20.719	101.92	1.689
137.92	3.948	59.83	5.571	100.68	4.718	136.36	3.365
191.33	6.047	78.62	6.876	194.8	9.699	212.42	14.97
237.93	9.663	143.07	15.412	278.88	22.662	274.39	11.034
270.14	9.073	232.08	20.01	325.45	4.249	373.56	3.553
394.67	25.129	303.32	170.409	401.8	15.098	410.02	21.531
445.82	56.883	343.28	9.331	486.2	11.502	540.62	31.225
468.94	1.045	444.5	3.57	559.71	1.159	603.42	66.162
491.51	18.413	458.22	8.469	597.45	34.004	638.96	79.998
540.92	56.444	489.18	54.046	636.96	38.822	657.2	23.663
574.88	10.264	537.42	33.136	695.83	36.289	698.24	9.09
615.61	106.966	574.94	58.266	709.98	210.757	784.68	92.779
640.51	75.073	644.25	15.352	792.96	62.685	816.88	0.956
718	9.484	717.56	144.373	847.37	48.53	851.07	15.901
739.27	2.152	748.13	37.817	922.52	21.445	921.06	18.789
778.21	87.088	767.97	51.225	959.54	18.257	1023.75	4.458
859.9	1.878	810.37	40.622	993.25	6.619	1046.13	12.879
864.21	10.461	883.48	2.835	1022.22	1.735	1060.38	4.032
990.21	31.023	890.95	1.209	1115.94	59.504	1088.57	3.587
995.88	16.016	996.32	15.67	1151.45	6.42	1104.96	39.463
1019.63	3.897	1015.4	0.958	1173.9	15.596	1119.47	40.544
1031.85	0.853	1041.92	0.328	1202.64	2.811	1292.78	36.308
1060.12	3.944	1050.01	24.093	1378.16	4.932	1344.75	85.261
1158.2	180.589	1175.72	122.785	1401	16.453	1415.77	29.198
1195.13	20.059	1211.34	135.602	1439.19	12.089	1480.79	65.505
1216.74	6.837	1251.25	35.743	1676.19	20.987	1518.72	63.402
1286.76	3.393	1386.65	1135.649	1687.44	48.955	1611.26	152.312
1396.29	14.317	1415.46	925.909	1948.7	107.352	1734.6	370.656
1434.93	23.589	1429.93	546.716	2273.66	749.246	2190.44	442.294
1471.19	40.858	1480.73	236.204	3165.67	35.214	3207.76	8.223
1549.93	168.048	1564.61	21.755	3169.22	20.345	3245.8	0.108
1581.83	115.491	1590.38	12.324	3219.66	3.276	3267.77	7.148
1646.88	495.098	1670.94	1.707	3250.62	1.414	3280.79	9.167
1652.01	21.062	1717.61	256.315	3510.21	164.943	3581.45	234.362
1731.66	255.926	2045.75	5605.121	3609.96	110.294	3690.1	88.555
3220.88	0.351	3215.32	0.436				
3231.74	0.556	3225.34	0.763				
3238.31	8.825	3233.41	1.793				
3246.69	7.209	3242.73	1.674				
3576.94	264.261	3545.32	212.973				
3685.74	70.785	3672.93	112.291				

Table S13: Harmonic frequencies (cm^{-1}) and intensities (km mol^{-1}) for transition states within H transfer pathway,calculated at the CAM-B3LYP/6-311+G* level (TS1c to TS4c).

TS1c		TS2c		TS3c		TS4c	
Frequency	Intensity	Frequency	Intensity	Frequency	Intensity	Frequency	Intensity
-561.21	430.831	-153.16	44.565	-234.54	98.744	-99.68	2.362
112.47	3.51	104.64	8.25	101.9	1.661	122.36	5.53
132.88	2.739	158.66	29.978	130.53	3.543	184.51	6.273
241.29	3.975	214.35	0.499	236.22	8.782	200.33	0.872
256.27	9.211	256.87	8.676	252.69	3.033	333.52	1.91
365.54	4.903	370.09	5.49	372.66	0.554	356.65	4.84
396.29	5.736	380.64	1.366	406.47	1.234	426.19	2.25
416.22	1.953	430.65	3.71	410.05	12.417	470.64	5.4
494.95	26.239	468.97	7.903	488.74	35.862	543.62	6.922
552.46	15.761	547.78	18.62	519.65	126.83	559.27	11.165
564.01	4.48	569.92	12.518	553.47	12.917	617.46	99.438
622.18	53.464	625.28	11.094	564.54	15.223	658.14	19.043
671.5	3.016	652.84	14.802	657.53	36.707	700.68	6.323
690.54	26.576	703.59	9.217	684.3	23.426	723.44	44.906
731.43	22.678	749.76	42.9	727.92	41.219	758.98	14.461
797.18	26.997	794.63	63.193	769.08	7.249	776.57	85.363
800.69	48.336	874.49	15.373	793.92	42.28	783.33	64.14
866.84	2.657	890.8	29.284	861.3	72.346	875.62	9.073
899.45	8.729	912.3	14.305	881.51	31.626	889.14	1.067
1012.39	3.843	1019.28	0.777	952.85	301.895	978.3	3.985
1049.23	89.845	1031.71	38.304	1016.11	1.667	1028.83	0.131
1052.1	2.472	1045.81	2.238	1051.31	0.654	1041.18	5.936
1079.59	87.401	1074.32	28.834	1054.98	9.63	1066.39	7.438
1097.51	46.112	1096.56	34.668	1101.62	184.614	1115.64	24.163
1150.82	78.909	1181.69	41.953	1157.96	84.301	1179.8	30.335
1193.99	226.717	1205.53	33.275	1206.26	185.742	1192.28	20.43
1228.55	158.014	1259.86	332.893	1243.67	335.558	1210.42	290.22
1273.79	23.379	1330.74	72.877	1277.02	131.205	1322.19	5.57
1319.29	7.193	1340.25	161.016	1320.4	72.423	1338.21	57.79
1372.2	32.432	1405.36	14.734	1401.61	13.623	1360.88	0.569
1414.94	249.974	1437.58	55.467	1444.91	24.819	1455.53	1.696
1468.94	55.693	1476.81	89.968	1488.24	37.677	1474.44	0.454
1503.91	35.691	1506.32	13.517	1517.47	91.054	1497.31	4.885
1607.17	64.997	1560.38	1.798	1598.06	112.285	1577.3	20.736
1630.76	155.17	1605.39	511.616	1661.48	299.05	1621.75	27.764
1754.89	338.416	1633.94	256.015	1720.23	341.874	1815.53	192.704
3226.64	0.526	3227.88	5.169	3223.99	0.578	3217.99	1.232
3238.05	3.61	3235.46	3.905	3239.31	0.325	3227.05	0.939
3243.25	12.766	3242.46	15.628	3242.44	8.16	3232.68	2.029
3249.55	4.747	3247.69	4.273	3253.18	7.129	3244.04	1.848
3424.96	39.493	3509.87	24.603	3473.39	68.723	3519.7	25.258
3462.88	126.599	3670.32	139.393	3639.29	336.768	3576.38	238.195

Table S14: Harmonic frequencies (cm^{-1}) and intensities ($km\ mol^{-1}$) for transition states within H transfer pathway,calculated at the CAM-B3LYP/6-311+G* level (TS5c to TS8c).

TS5c		TS6c		TS7c		TS8c	
Frequency	Intensity	Frequency	Intensity	Frequency	Intensity	Frequency	Intensity
-209.11	74.444	-157.77	2.142	-591.44	99.827	-495.19	37.39
114.57	1.445	106.47	1.262	106.44	5.975	104.67	3.259
155.8	7.078	173.96	2.636	154.58	5.238	137.03	4.448
230.3	4.343	194.29	1.035	244.02	12.115	241.9	2.351
243.95	6.321	332.67	1.242	327.63	11.864	263.75	2.366
360.2	1.671	347.45	0.815	344.2	45.099	317.27	116.32
406.05	22.682	454.83	20.191	367.46	113.772	406.26	75.07
439.74	3.431	470.52	1.671	452.2	58.383	430.06	19.642
484.99	142.506	542.8	10.836	488.95	20.542	459.14	16.036
507.18	9.772	557.42	4.533	515.4	3.101	520.19	27.2
536.43	2.093	631.28	85.527	550.96	1.493	541.52	4.512
565.59	28.142	646.79	35.4	577.01	5.514	567.91	5.062
663.88	27.954	651.76	50.05	635.55	13.986	617.11	1.853
683.69	21.424	728.51	8.456	743.14	28.632	742.32	2.455
731.36	58.68	735.79	30.982	754.57	30.462	781.45	97.635
770.45	8.493	771.99	59.784	764.87	0.64	788.42	16.241
787.1	40.024	776.91	40.151	785.68	67.161	873.48	0.141
850.18	188.895	872.88	6.301	877.42	4.913	889.07	64.095
874.06	19.392	897.79	0.121	888.85	0.867	924.22	73.778
919.44	206.177	981.48	2.508	983.9	29.65	949.77	59.652
1011.51	0.796	1026.67	8.613	996.18	35.78	1011.35	2.945
1050.69	0.967	1034.73	0.197	1019.56	57.798	1015.41	9.465
1054.44	3.863	1066.42	3.464	1035.39	0.11	1032.03	38.727
1080.28	186.129	1113.17	51.295	1036.3	4.625	1049.18	12.697
1190.09	72.219	1156.94	39.591	1146.17	7.856	1049.39	4.737
1207.94	210.387	1183.92	278.965	1194.72	11.14	1171.48	3.274
1255.73	422.093	1199.95	56.206	1215.66	72.493	1198.72	17.81
1286.74	21.323	1310.9	3.548	1275.42	39.673	1291.42	13.295
1344.34	56.547	1332.31	37.76	1310.81	45.453	1327.52	29.81
1400.17	84.355	1352.59	7.703	1353.19	5.892	1362.47	1.66
1450.84	2.515	1442.36	3.54	1435.65	7.839	1429.64	33.574
1491.77	13.666	1450.08	10.217	1449.43	13.831	1453.2	16.911
1506.79	64.026	1483.04	3.283	1466.67	52.917	1479.35	24.822
1580.13	189.329	1583.15	19.458	1528.73	12.91	1577.34	16.906
1670.53	143.845	1607.08	38.501	1584.66	3.01	1641.28	34.688
1707.43	251.923	1793.21	209.539	1633.54	74.983	1666.26	27.63
3223.49	0.534	3220.22	2.51	3227.32	0.227	3227.51	0.545
3241.07	0.816	3228.52	0.229	3237.74	0.457	3239.02	1.252
3244.44	5.113	3239.53	5.056	3244.68	5.103	3248.18	5.387
3260.93	9.245	3245.19	2.885	3249.52	5.109	3257.55	7.167
3483.95	88.346	3480.23	2.765	3603.52	267.16	3615.22	344.887
3657.2	340.269	3562.34	242.508	3728.72	213.592	3727.04	125.664

SV Geometries of *aci*-Rearranged ONA^+ Conformers

Table S15: Cartesian coordinates for INT1c and INT2c optimized at the CAM-B3LYP/6-311+G* level.

	INT1			INT2		
	X	Y	Z	X	Y	Z
C	0.449526355	-1.064457099	-0.106129515	0.489853217	-1.075049429	-0.063877767
C	-0.217371430	0.247128077	-0.028209220	-0.233241464	0.206305985	0.062682229
C	1.887008976	-1.025524606	0.031323576	1.930371111	-0.968510360	-0.129272445
C	2.580185685	0.152705856	0.103758358	2.580742000	0.231358556	-0.043786714
C	0.515392238	1.431186349	0.004608000	0.461551556	1.411038672	0.167034751
C	1.898409011	1.383052701	0.068869006	1.845956747	1.418445665	0.122194023
N	-0.064000765	-2.222006677	-0.383216473	0.007867238	-2.271766994	-0.131240543
H	-1.916692625	-1.131455864	1.140988478	-1.843950678	1.908852697	-0.795880843
H	-1.059982013	-2.212145024	-0.604025209	-1.008204462	-2.324893488	-0.075047630
N	-1.597514246	0.375616426	-0.005774473	-1.611090488	0.210771364	0.070897739
O	-2.353816744	-0.685755531	0.391908496	-2.281719110	1.383840288	-0.103835451
O	-2.200586839	1.344353762	-0.335276875	-2.301046535	-0.743921060	0.280883363
H	2.385672073	-1.986866489	0.015916746	2.461694406	-1.906343994	-0.236626150
H	3.660691242	0.142167392	0.180143196	3.662431460	0.271252158	-0.081929918
H	2.454769184	2.311181961	0.120252251	2.366930667	2.362017580	0.234444951
H	-0.005904408	2.380387418	0.028715710	-0.063988007	2.339722547	0.352347930

Table S16: Cartesian coordinates for INT3c and INT4c optimized at the CAM-B3LYP/6-311+G* level.

	INT3			INT4		
	X	Y	Z	X	Y	Z
C	0.426543171	-1.055497790	0.037242535	0.460720643	-1.075100653	0.019408929
C	-0.228721671	0.238509438	-0.088118611	-0.220802038	0.236883209	-0.013948157
C	1.860496649	-1.014952423	0.065691061	1.908216792	-1.013039198	0.024345840
C	2.549253800	0.169585439	0.002089053	2.598680448	0.166276371	0.000503612
C	0.481260208	1.429770951	-0.146186360	0.517158361	1.422672751	-0.037470144
C	1.866960824	1.392527286	-0.106781707	1.900308943	1.386535769	-0.030381574
N	-0.302424772	-2.124278129	0.198909539	-0.035247018	-2.266397098	0.045258386
H	-1.742088302	1.797547952	0.966887628	-3.251776904	-0.471100931	-0.014519291
H	0.263355533	-2.969928290	0.280656838	-1.050926797	-2.303575823	0.041997362
N	-1.631008864	0.271169827	-0.183192069	-1.588159525	0.392623817	-0.024311099
O	-2.282496671	1.363438788	0.283606308	-2.314409096	-0.747305371	-0.004914850
O	-2.302007964	-0.563100199	-0.665413626	-2.177408669	1.440447522	-0.048771466
H	2.389603068	-1.957373800	0.153210445	2.408809637	-1.973061089	0.048821130
H	3.632396729	0.165383247	0.023885197	3.681789509	0.165626482	0.005280509
H	2.423918972	2.317633334	-0.189023697	2.447490880	2.321511027	-0.049549334
H	-0.023471161	2.376095155	-0.304449913	-0.004291532	2.370664896	-0.061845746

Table S17: Cartesian coordinates for INT5c and INT6c optimized at the CAM-B3LYP/6-311+G* level.

	INT5			INT6		
	X	Y	Z	X	Y	Z
C	0.440932614	-1.075204253	-0.003579602	0.493221018	-1.077002573	-0.016327737
C	-0.223776125	0.232875767	0.015727799	-0.245780798	0.188879146	0.001161301
C	1.884251275	-1.030949592	-0.009620021	1.930399960	-0.954832820	0.012573528
C	2.581890478	0.144700119	0.003080894	2.556037366	0.261463217	0.025829069
C	0.513739876	1.415392894	0.028832880	0.415371433	1.414766777	0.005232376
C	1.896463003	1.373010581	0.022759125	1.800057646	1.447275861	0.018712160
N	-0.236640978	-2.179975058	-0.013755833	-0.134952252	-2.208053790	-0.089755256
H	-3.275628636	-0.404027430	0.015966088	-3.159703118	1.262994478	-0.057624631
H	0.382279970	-2.992523750	-0.026966153	0.514685624	-2.996317878	-0.097471040
N	-1.605914472	0.397129256	0.021881597	-1.633243634	0.175508712	0.010070952
O	-2.344935315	-0.707193468	0.009830040	-2.194735388	1.404868910	-0.105991002
O	-2.157057965	1.463816605	0.036890151	-2.345157135	-0.771112099	0.107316782
H	2.406442408	-1.981264603	-0.024550621	2.507143481	-1.873181571	0.012708498
H	3.665145925	0.134687767	-0.001897893	3.637808193	0.316582462	0.042678809
H	2.452126310	2.302643432	0.033289933	2.302214683	2.406892956	0.030940300
H	-0.002898346	2.365804430	0.043634875	-0.146483167	2.337676818	0.012861320

Table S18: Cartesian coordinates for INT7c optimized at the CAM-B3LYP/6-311+G* level.

	INT7		
	X	Y	Z
C	0.520874713	-1.075606470	0.040223201
C	-0.236186662	0.192533577	0.009260969
C	1.961109094	-0.935313278	0.029593040
C	2.578849779	0.284796346	-0.006890387
C	0.424471626	1.421809391	-0.027596426
C	1.809516143	1.460896281	-0.035434153
N	0.075539543	-2.286291499	0.075092840
H	-3.164777121	1.201473565	-0.005679151
H	-0.940092459	-2.359664445	0.081816455
N	-1.605136902	0.167866166	0.015522866
O	-2.204389503	1.378037908	-0.014361365
O	-2.302312120	-0.816507221	0.044525287
H	2.518022742	-1.863983540	0.052579411
H	3.659981663	0.350106373	-0.013973152
H	2.302632418	2.425314355	-0.064556776
H	-0.142761983	2.341777477	-0.050601066

SVI Supplemental Figures of Experimental Data

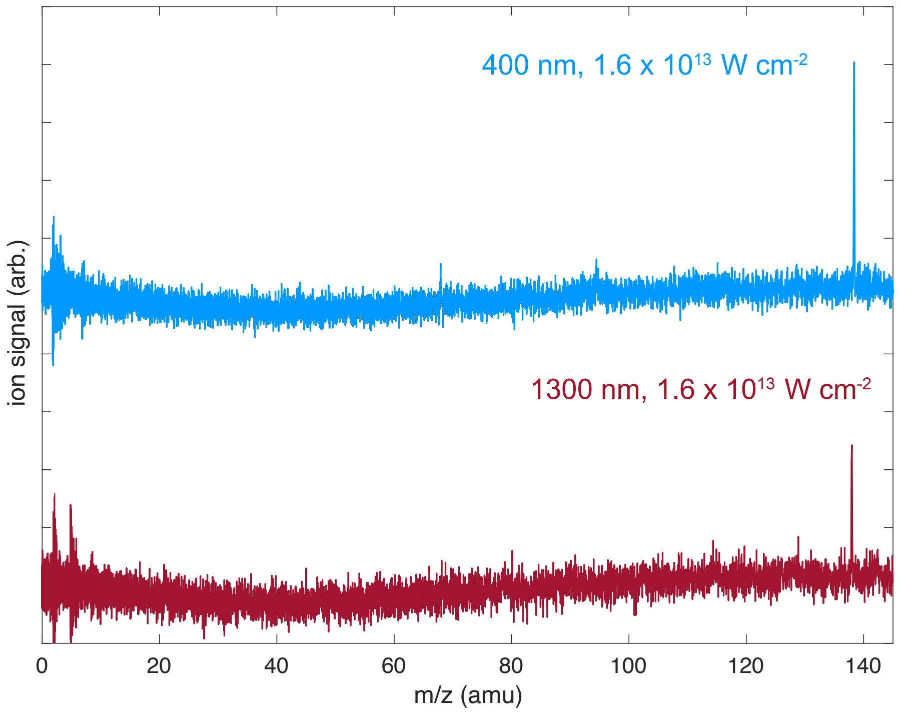

Figure S1: SFI-MS of ONA taken at the threshold intensity of 1.6×10^{13} W cm^{-2} for ionization. In the FTRMS measurements, the intensity of the 400 nm probe was kept at half of this value (8×10^{12} W cm^{-2}) by lowering the measured average power by a factor of 2.

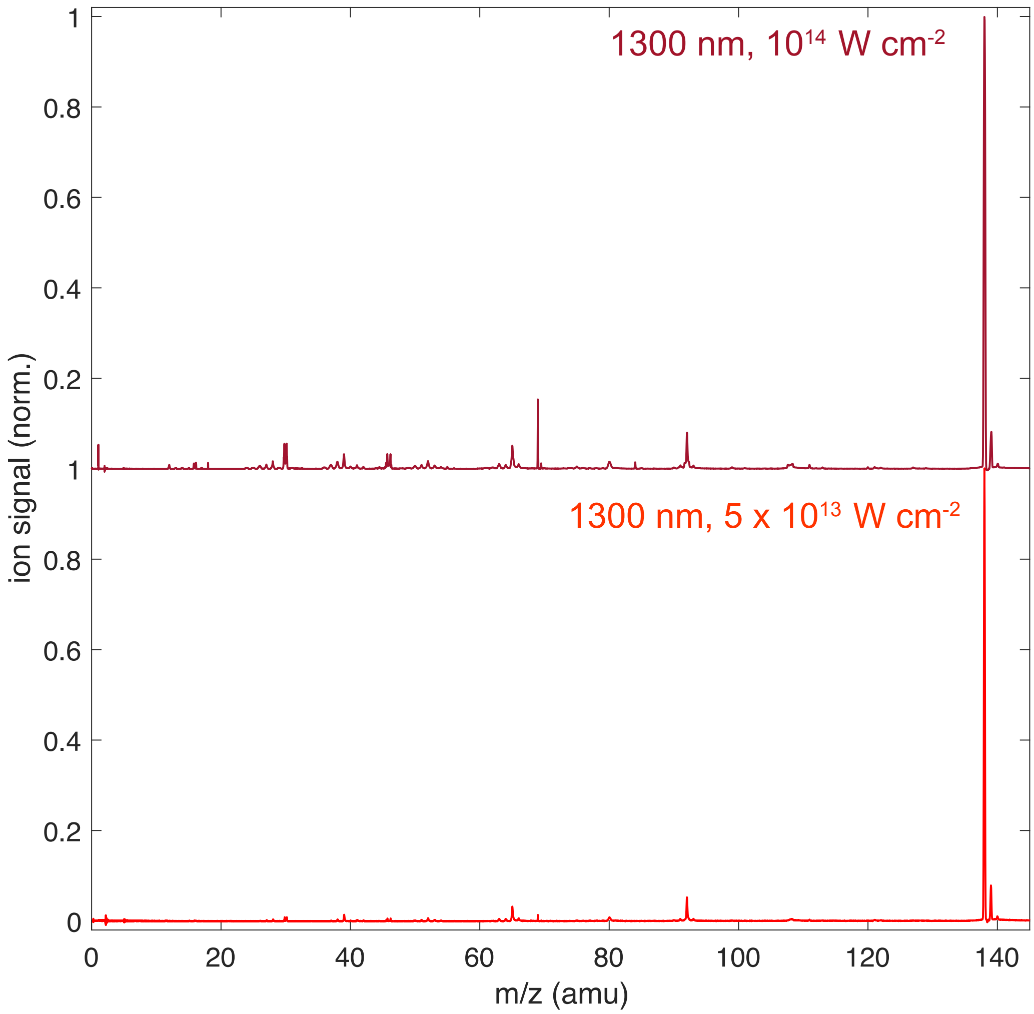

Figure S2: SFI-MS of ONA taken at 1300 nm with intensity 10^{14} W cm^{-2} (top) and 5×10^{13} W cm^{-2} (bottom). At the lower intensity, the yields of doubly charged species are extremely low.

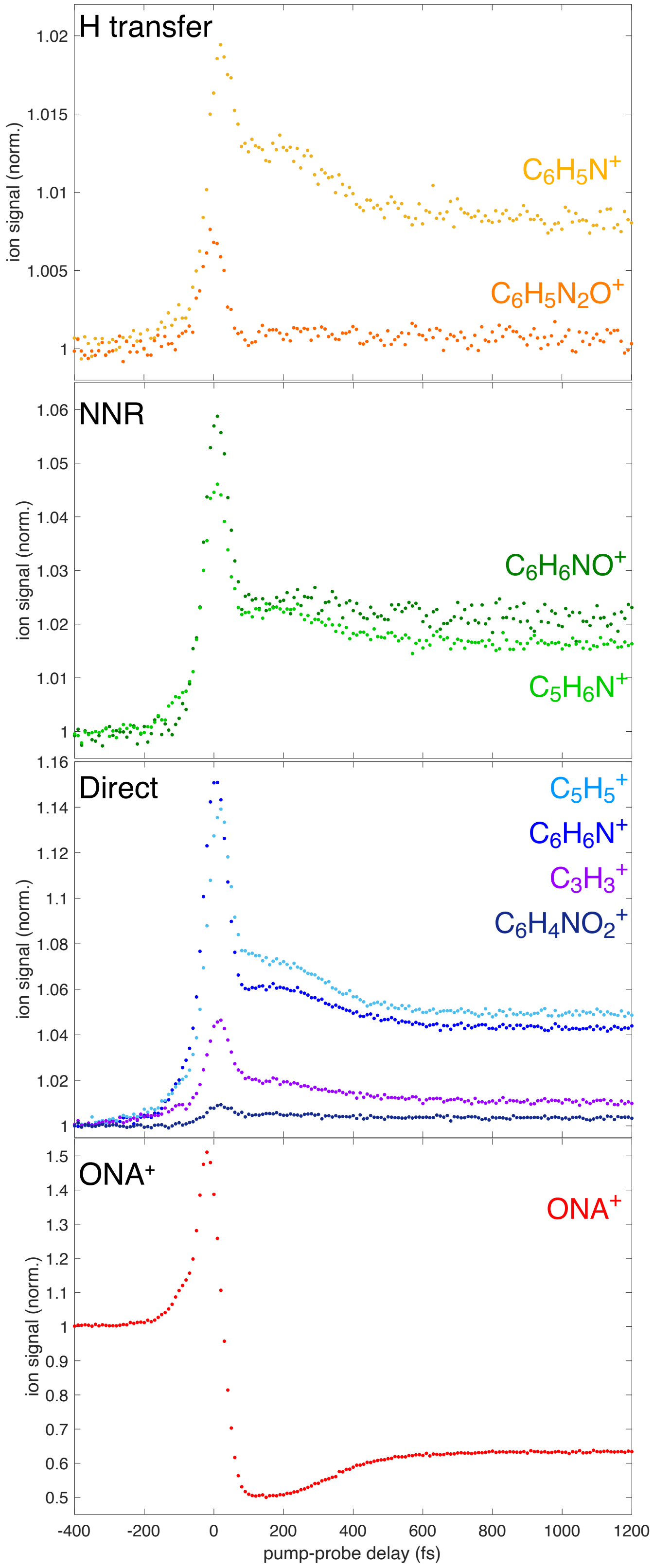

Figure S3: FTRMS measurements of ONA using a pump intensity of 5×10^{13} W cm^{-2}. Similar fragment ion dynamics are observed as those in Figure 4, where 10^{14} W cm^{-2} pump intensity was used.

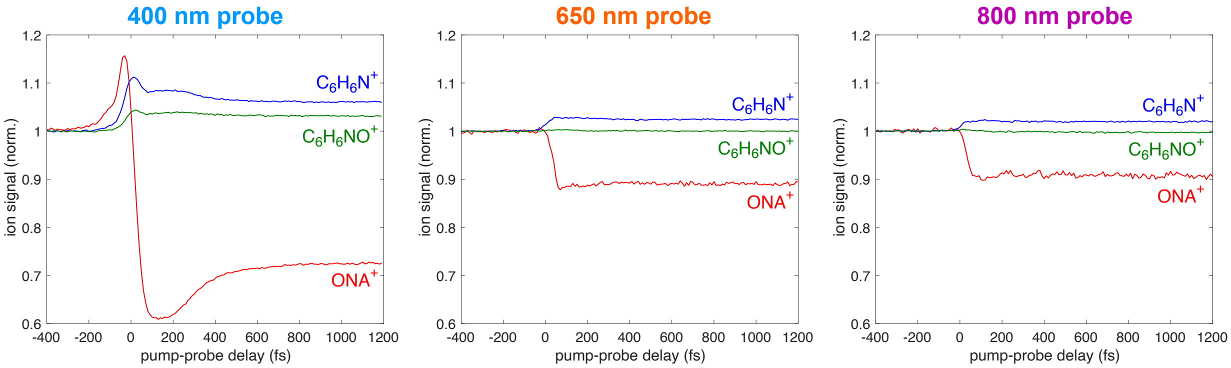

Figure S4: Transients for each probe wavelength.

SVII Alternative method for the estimation of relaxation times

Figure S5a shows the atom labels in ONA cation. Figure S5b shows the evolution of the C2–C5 bond length along one representative AIMD trajectory out of the 105 trajectories that were calculated. The black line represents the recorded bond length; the red and blue lines represent the upper and lower envelopes of the trajectory amplitude as the magnitude of its analytic signal. The analytic signal is found using the Hilbert transform as implemented in the *hilbert* function of MATLAB. The trajectory shows an oscillatory behaviour that is strongly damped in regions around the "equilibrium value", the C2–C5 equilibrium bond length in the case of Figure S5b (magenta dotted line). We use the broad term "equilibrium value" because this kind of oscillatory behavior is present in all the degrees of freedom no matter if they were bond lengths, angles, or dihedrals. Considering this behavior, we estimate the relaxation time as the time corresponding to the minimum of the upper envelope for each trajectory (identical results are obtained if the maximum of the lower envelope is considered instead). It can be seen that the relaxation time for the trajectory shown in Figure S5b is 315 fs. By performing this procedure for the C2–C5 bond length along the 105 trajectories, we obtained an average of 233±131 fs to reach the equilibrium bond length. Figure S5c shows a histogram of the relaxation times obtained for this bond length. Additionally, we

performed the same analysis considering the C1–N7 bond length (Figure S6), C1–N7–H8 angle (Figure S7), and H9–O11 bond length (Figure S8), and the results are summarized in Table S19.

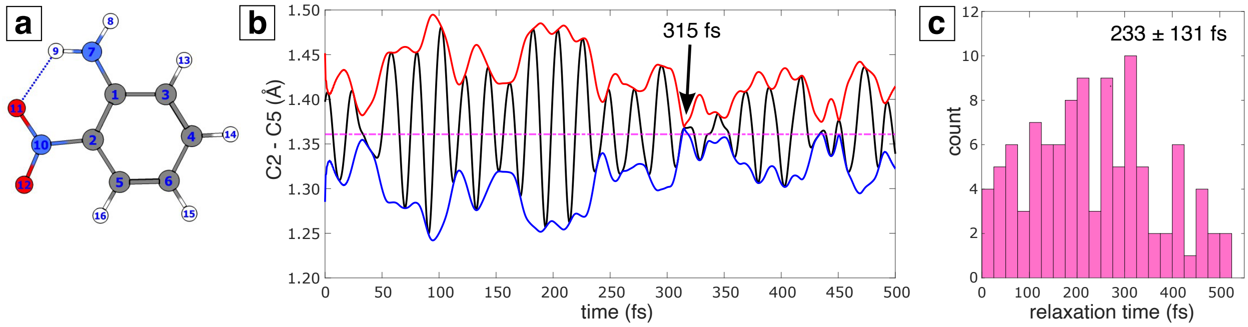

Figure S5: (a) ONA cation (S_0 geometry) with the corresponding atom labels. (b) C2–C5 bond length along a sample AIMD trajectory (black line), upper and lower amplitude envelopes (red and blue lines respectively), and C2–C5 equilibrium bond length for ONA cation (magenta dotted line). (c) Histogram for the C2–C5 bond relaxation times obtained from 105 AIMD trajectories (average and standard deviation shown). Results were obtained at the CAM-B3LYP/6-311G(d) level.

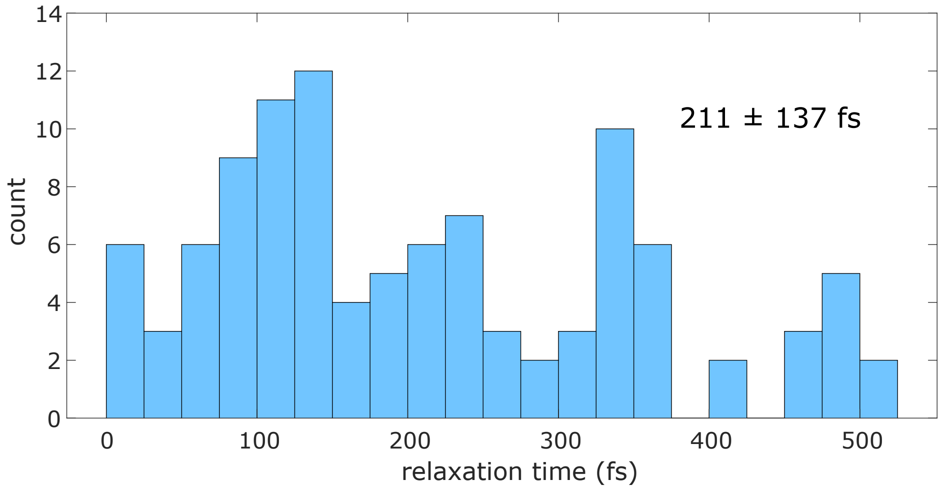

Figure S6: Histogram for the C1–N7 bond relaxation times obtained from 105 AIMD trajectories (average and standard deviation shown). Trajectories were obtained at the CAM-B3LYP/6-311G(d) level.

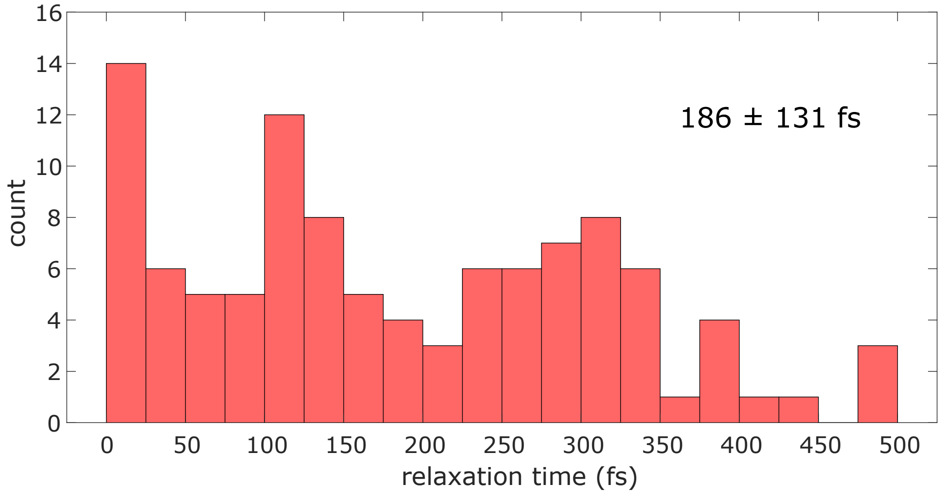

Figure S7: Histogram for the C1–N7–H8 angle relaxation times obtained from 105 AIMD trajectories (average and standard deviation shown). Trajectories were obtained at the CAM-B3LYP/6-311G(d) level.

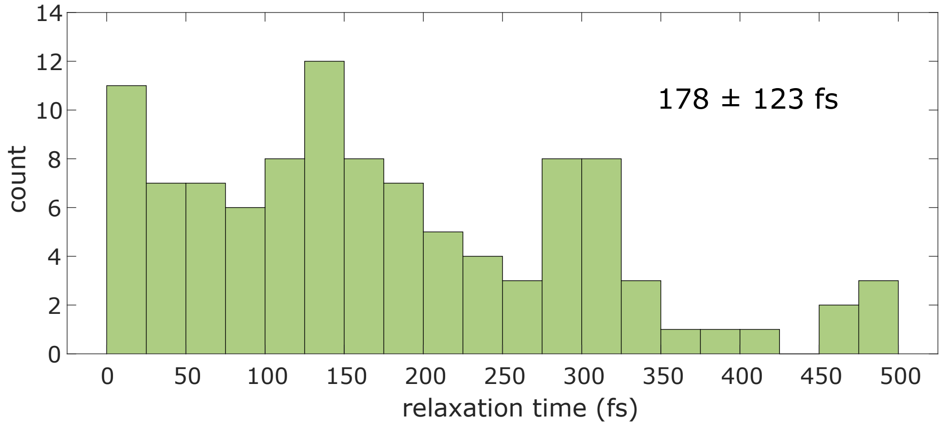

Figure S8: Histogram for the H9–O11 bond relaxation times obtained from 105 AIMD trajectories (average and standard deviation shown). Trajectories were obtained at the CAM-B3LYP/6-311G(d) level.

Table S19: Mean relaxation time and the corresponding standar deviation (SD) over 105 AIMD trajectories for different degrees of freedom (DOF).

DOF	mean relaxation time (fs)	SD (fs)
C2-C5	233	131
C1-N7	211	137
C1-N7-H8	186	131
H9-O11	178	123
